# Sensitization of Non‐M3 Acute Myeloid Leukemia Blasts to All‐*Trans* Retinoic Acid by the LSD1 Inhibitor Tranylcypromine: TRANSATRA Phase I Study

**DOI:** 10.1111/ejh.14426

**Published:** 2025-06-03

**Authors:** Michael Kruszewski, Claudia Schmoor, Tobias Berg, Usama‐Ur Rehman, Marcus Schittenhelm, Katharina Götze, Andrea Kündgen, Caroline Pabst, Tobias Ma, Anna Frey, Julia Stomper, Dietmar Pfeifer, Eric Metzger, Johannes Jung, Kevin Moschallski, Johanna Thomas, Gesine Bug, Justus Duyster, Manfred Jung, Roland Schüle, Ralph Wäsch, Olga Grishina, Michael Lübbert

**Affiliations:** ^1^ Department of Hematology, Oncology and Stem Cell Transplantation Medical Center—University of Freiburg Freiburg Germany; ^2^ Clinical Trials Unit Medical Center—University of Freiburg Freiburg Germany; ^3^ Centre for Discovery in Cancer Research, Department of Oncology McMaster University Hamilton Ontario Canada; ^4^ Escarpment Cancer Research Institute, Hamilton Health Sciences McMaster University Hamilton Ontario Canada; ^5^ Department of Medicine II Hematology/Oncology Goethe University Frankfurt/Main Germany; ^6^ Department of Oncology and Hematology Cantonal Hospital St. Gallen St. Gallen Switzerland; ^7^ Department of Internal Medicine II, Hematology, Oncology, Clinical Immunology, and Rheumatology University Hospital Tübingen Tübingen Germany; ^8^ Medical Department of Hematology and Oncology Technical University of Munich, Klinikum Rechts der Isar Munich Germany; ^9^ German Cancer Consortium (DKTK) Munich Germany; ^10^ German Cancer Research Center (DKFZ) Heidelberg Germany; ^11^ Department of Hematology, Oncology and Clinical Immunology Düsseldorf University Hospital Düsseldorf Germany; ^12^ Department of Medicine V, Hematology, Oncology and Rheumatology University Hospital Heidelberg Heidelberg Germany; ^13^ Department of Pathology Medical Center—University of Freiburg Freiburg Germany; ^14^ German Cancer Consortium (DKTK) Freiburg Germany; ^15^ Institute of Pharmaceutical Sciences University of Freiburg Germany; ^16^ Department of Urology and Center for Clinical Research Medical Center—University of Freiburg Freiburg Germany

**Keywords:** CD38, chromatin, differentiation, histone demethylase, LSD1, myelodysplastic syndrome

## Abstract

The treatment of elderly, nonfit acute myeloid leukemia (AML)/MDS patients with relapsed/refractory (R/R) disease remains challenging. As histone demethylase LSD1 (KDM1A) is a rational therapeutic target in AML, we conducted a phase I trial (“rolling‐six design”) with the LSD1 inhibitor tranylcypromine (TCP, dose levels [DL] 20, 40, 60, 80 mg p.o. d1‐28) combined with fixed‐dose ATRA (45 mg/m^2^ p.o. d10‐28) and low‐dose cytarabine (LDAC, 40 mg s.c. d1‐10). The primary endpoint was dose‐limiting toxicity (DLT) in the first 28 days of treatment. The aim was the determination of the maximum tolerated TCP dose (MTD). Twenty‐three patients with AML and 2 with MDS were accrued. TCP was administered for a median of 39.5 days (range: 11–228). No DLTs were observed at any DL; MTD could not be established. No differentiation syndrome occurred. Two patients attained a PR; SD was achieved in 10 of 22 evaluable patients. Median OS was 62 days (range: 14–325). Accompanying studies included pharmacokinetics, serial determinations of fetal hemoglobin (HbF), detection of CD38 upregulation with treatment, as well as transcriptome changes in purified blood blasts over time. In conclusion, the combination of TCP with ATRA and LDAC was well feasible, even at the highest DL. Hence, studies with more potent LSD1 inhibitors appear warranted.

**Trial Registration:** German Clinical Trials Register (DRKS): DRKS00006055. For further Information see https://drks.de/search/en/trial/DRKS00006055

## 1 Introduction

Treatment of older unfit patients with acute myeloid leukemia (AML) who are unable to tolerate intensive chemotherapy remains challenging [1, 2]. While hypomethylating agents (HMAs) offer a less intensive alternative, the development of resistance often leads to poor long‐term outcomes [3, 4, 5]. This underscores the urgent need for innovative therapies that can effectively address relapsed/refractory AML in these vulnerable patients.

LSD1 (lysine‐specific demethylase 1A, KDM1A) is emerging as a novel epigenetic therapeutic target in AML. LSD1 demethylates mono‐ or dimethylated lysines 4 and 9 on histone H3 [6]. RARA expression is diminished in AML, and inhibition of LSD1 has been shown to reactivate the retinoic acid (RA)‐induced differentiation pathways [7, 8]. At present, all‐*trans* RA (ATRA) has exclusively been established as a treatment of acute promyelocytic leukemia (APL). Sensitization of non‐APL AMLs to ATRA is thought to create additional treatment options, especially for unfit patients. In the DECIDER phase II trial, the combination of an HMA (decitabine) and ATRA has resulted in an in vivo synergism, demonstrating that the addition of ATRA to decitabine significantly prolonged overall survival and remission rates compared to decitabine alone in elderly, unfit newly diagnosed AML patients, without added toxicity [9].

In this study we repurposed tranylcypromine (TCP, *trans*‐2‐phenylcyclopropylamine) as an LSD1 inhibitor. This drug has a well‐established safety profile, having been used as an antidepressant for over 50 years. It acts as an irreversible inhibitor of LSD1 and has been shown to sensitize cells to ATRA and induce differentiation [8, 10, 11]. The addition of LDAC is as a “debulking” backbone therapy and to prevent differentiation syndrome. In accompanying studies we serially captured patients' health‐related quality of life, anxiety and depression, dynamics of fetal hemoglobin (HbF) expression as a potential predictive biomarker for erythroid differentiation during hypomethylating treatment [4, 12] and serial CD38 expression on AML blasts, a known marker of ATRA‐induced upregulation and therapeutic target [13].

This phase I trial investigates the combination of TCP, ATRA, and LDAC in patients with relapsed/refractory (R/R) AML or high‐risk MDS for whom standard treatment options are limited.

## 2 Patients and Methods

### 2.1 Study Design and Endpoints

TRANSATRA (*TRAN*ylcypromine *S*ensitization to *ATRA*) is a prospective single arm, open‐label phase I/II trial conducted at six academic centers in Germany (Düsseldorf, Frankfurt, Freiburg, Heidelberg, München, Tübingen).

The goal of the phase I part of the study was to determine the maximum‐tolerated dose (MTD) and the recommended phase II dose (RP2D) of TCP. The rolling‐six phase I design, a modification of the standard 3 + 3 design, was used to determine the MTD of TCP. For detailed specification see Skolnik et al., 2008 [14]. Four dose levels (DL) of TCP were examined.

The primary endpoint of the phase I part of the study was dose‐limiting toxicity (DLT) during the first cycle. DLT was defined as drug‐related toxicity resulting in dose reduction or treatment discontinuation of TCP. The MTD of TCP was defined as the highest level that results in a DLT in treatment cycle one in fewer than two in six patients who fulfill the minimum safety evaluation requirements.

The secondary endpoint of the phase I part of the study was safety in terms of adverse events (AEs) and serious adverse events (SAEs). Toxicities were described by system organ classes (SOCs) and preferred terms (PTs) using the *Medical Dictionary for Regulatory Activities* (MedDRA). The phase II part of the trial was to evaluate the efficacy. The primary endpoint was objective best response. Secondary endpoints were overall survival, evaluation of quality of life, and psychological distress. Due to slow accrual, phase II of the study was not performed. However, for patients enrolled in phase I, a descriptive analysis of efficacy endpoints was performed for all patients.

### 2.2 Patients

To be eligible for the trial, patients had to have AML (WHO 2008 criteria) or intermediate or higher‐risk MDS/CMML (IPSS‐*R* > 3.0) and no standard treatment available (due to azanucleoside failure, refractoriness to standard chemotherapy, salvage chemotherapy or allogeneic stem cell transplantation, comorbidities, higher age). Detailed inclusion and exclusion criteria are provided in Table S3. Written informed consent was obtained according to international guidelines and local laws. The trial protocol was approved by the Ethics Committee of the University of Freiburg.

### 2.3 Treatment

Patients were treated with a combination of LDAC, ATRA, and TCP. LDAC was given s.c. at the beginning of every cycle, days 1–10, twice daily, with a fixed daily dose of 40 mg, to achieve cytoreduction and prevent differentiation syndrome by early “debulking”. ATRA was given p.o. twice daily with a fixed daily dose of 45 mg/m^2^ and administered continuously after day 10 of the first cycle, with a 9‐day break every four cycles. TCP was given depending on the patients' DL (20, 40, 60, 80 mg). TCP was administered p.o. twice daily. TCP was started with a total daily dose of 20 mg and increased by 10 mg every 4 days up to the targeted DL.

### 2.4 Quality of Life, Depression, and Anxiety Assessment

The Hospital Anxiety and Health‐Related Depression Scale (HADS) and the EORTC QLQ‐C30 (for health‐related quality of life) questionnaires were taken during screening and after the first cycle.

### 2.5 Immunohistochemical Quantification of CD38 in Bone Marrow Biopsies

Bone marrow biopsies were performed at screening, at the end of the first and second cycles, and the end of treatment (EOT). NACE (Naphthol‐AS‐D Chloroacetate‐esterase) staining on formalin‐fixed paraffin‐embedded bone marrow trephine biopsy samples was performed to visualize cellular morphology according to established protocols. Subsequently, immunohistochemical labeling of CD38 was carried out. Staining intensity was scored into five groups ranging from weak, weak/moderate, moderate, moderate/strong, strong by an in‐house pathologist.

### 2.6 Measurement of Fetal Hemoglobin (HbF)

HbF levels were measured by high‐performance liquid chromatography (HPLC) before treatment and after the end of the first cycle, and, where possible, after every following cycle [15]. Patients with HbF levels of 1% or lower were considered normal, while HbF levels exceeding 1% were considered elevated, in accordance with reference values for the Medical Center—University of Freiburg—Central Laboratory.

### 2.7 Plasma Levels

Blood samples for pharmacokinetic studies were collected at designated intervals on days 10, 20, 28, and 56 in the morning before and 2 h after TCP intake. Samples were analyzed by an external laboratory (Medizinisches Labor Bremen).

### 2.8 NGS Profiling

Mutational profiling by NGS was performed before treatment on bone marrow mononuclear cells. A Qiagen Custom Pro Myeloid Panel was used to screen for mutations in 73 genes and specific hotspots.

### 2.9 RNA Sequencing

RNA was extracted from CD34+ and/or CD117+ enriched blast population from the peripheral blood of eight patients prior to treatment, from three of them also during treatment at different timepoints. Read quality was checked with FastQC (≥ 95% > Q30), alignment to the reference genome GRCh38/hg38 was performed with STAR aligner [16] and read‐counting was performed with featureCounts [17]. Each sample counts file was then fed to DESeq2 [18] to obtain the results file for treated samples vs. screening timepoint. Differentially expressed genes (DEG)s were found by filtering genes with adjusted *p* value < 0.05 and Log2 fold change > abs (0.6). The result file is ranked using sign[log2fold]*Log10[p‐adj] as suggested by Galaxy FGSEA tutorial before using them as input for FGSEA on the MSigDB Hallmark pathways database. All analysis steps till here are calculated on the European Galaxy instance [19]. The results files were further sorted, filtered, and plotted (Heatmap and Volcano plot) using Pandas, scikit, Matplotlib and the Seaborn library of Python. The result file is ranked using sign[log2fold]*Log10[p‐adj] [20]. Expression values are displayed in *z*‐score on the heatmap.

### 2.10 Statistical Methods

For the safety analysis, patients who started treatment with TCP were included, and patients were analyzed according to the TCP level achieved. For the descriptive analysis of the efficacy endpoints objective best response, overall survival, quality of life, and psychological distress, all patients were combined in one group.

Patient selection for translational research including CD38 assessment in bone marrow biopsies, HbF measurement, NGS profiling, and RNA sequencing was dependent on available material and time points of preservation.

To compare HbF% distribution, a Wilcoxon signed rank test (due to non‐normality) was used. To compare TCP serum levels between DL before and after intake, a Brown‐Forsythe ANOVA was used (due to unequal variances). Survival data was analyzed using a Kaplan–Meier method. Statistical analysis and computing were conducted with SAS9.4 and GraphPad Prism 10.

## 3 Results

### 3.1 Patient Characteristics

A total of 25 patients were enrolled in the phase I part of the trial (Table 1). Median age was 75 years (range: 62–84), with five patients being 80 or older. Twenty‐three of 25 (92%) patients had AML, most of them (69.6%) had relapsed disease, four patients (17.4%) had primary induction failure, and three patients (13.0%) were untreated. Previous HMA treatment failure was documented in 23 patients (92%).

**TABLE 1:** Patient characteristics.

Characteristics	*n* = 25
Sex, *n* (%)	
Male	14 (56.0)
Female	11 (44.0)
Age, year, median (range)	75 (62–84)
ECOG, *n* (%)	
0	1 (4.0)
1	14 (56.0)
2	10 (40.0)
Entity, *n* (%)	
AML	23 (92.0)
MDS	2 (8.0)
HCT comorbidity index (HCT‐CI), median (SD)	1.8 (2.04)
≥ 4, *n* (%)	4 (16.0)
Prior treatment lines, median (range)	2 (1–5)
Prior HMAs, *n* (%)	23 (92.0)
Blasts percentage, median (range)	
Peripheral blasts (*n* = 24)	15 (0.0–89.0)
Bone marrow blasts	46 (5.0–95.0)
AML—ELN 2010^a^, *n* (%)	*n* = 23
Favorable	5 (21.7)
Intermediate I/II	9 (39.1)
Unfavorable	9 (39.1)
MDS—IPSS‐R	*n* = 2
High‐Risk	2 (100.0)

Abbreviations: ECOG, Eastern Cooperative Oncology Group; AML, acute myeloid leukemia; MDS, myelodysplastic syndrome; HCT‐CI, Hematopoietic Cell Transplantation‐Comorbidity Index; HMA, hypomethylating agent; ELN, European LeukemiaNet; IPSS‐R, Revised International Prognostic Scoring System.

^a^ According to study protocol.

Most common mutations detected by NGS profiling were TET2 (*n* = 8), ASXL1 (*n* = 8) and DNMT3A (*n* = 6) (Figure S1).

### 3.2 Drug Exposure

**FIGURE 1:**
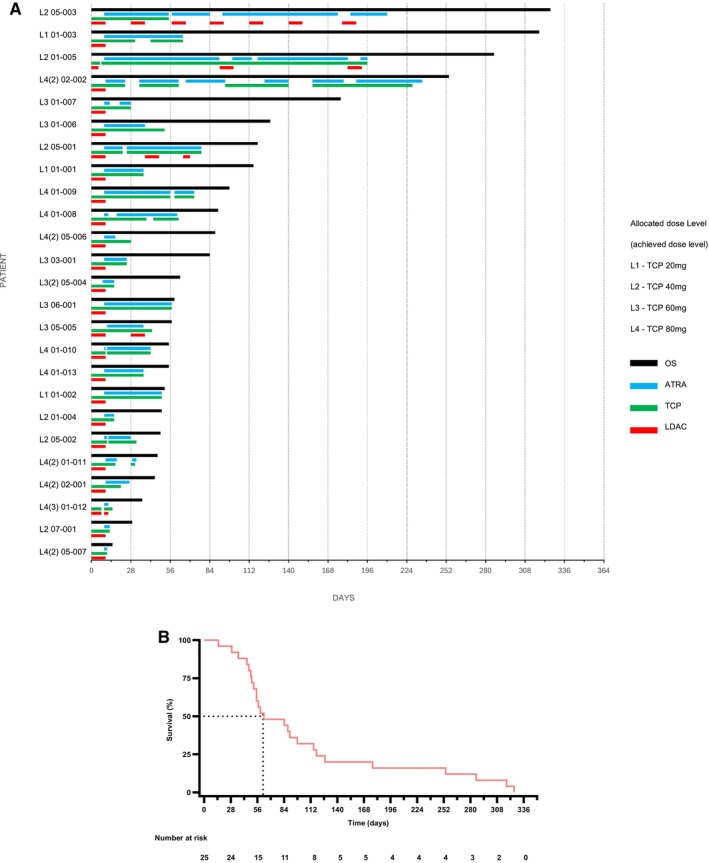
(A) Treatment exposure and overall survival. Overall survival depicted next to number of days of exposure to TCP, ATRA, and LDAC. For patient 01‐013 last documented intake dates of TCP and ATRA were taken. For patient 02‐001 last documented intake date of ATRA was taken. Exact dates of last intake are unknown for both patients. Interruptions are due to protocol, adverse events, or other reasons. The allocated dose level of each patient is depicted (L1‐4); if the allocated dose level was not reached, the highest achieved dose level is shown in parenthesis. (B) Kaplan–Meier plot. Overall survival rate estimated by Kaplan–Meier method. The median OS was 62 days. The number at risk is shown below the graph.

The number of patients with their respective allocated and achieved DL are shown in Figure S2. Patients received ATRA for a median of 28 days (range: 2–207). LDAC was administered for a median of 10 days (range: 10–192). Patients received TCP for a median of 39.5 days (range: 11–228), and seven patients received TCP for more than 56 days (Figure 1A). No DLT was observed in the first 28 days of treatment.

### 3.3 Maximum Tolerated Dose and the Recommended Phase II Dose

Twelve patients (three patients on each DL) fulfilled minimum safety evaluation requirements and were fully evaluable for MTD. No DLT was observed in the first 28 days of treatment on any of the four DLs. Nevertheless, as only three of 10 patients treated on the highest DL (80 mg) were fully evaluable, the MTD defined as the highest level that resulted in DLT in fewer than two in six patients in the first 28 days of treatment could not be established.

### 3.4 Safety and Tolerability

Overall, 214 AEs were observed. Twenty‐four of the 25 patients who started TCP treatment experienced at least one AE during the trial. The mean number of AEs per patient was 8.3, 9.8, 6.0, and 9.0 in DL 20, 40, 60, and 80 mg, respectively. Treatment with TCP was stopped in three patients due to an AE, all of them on lower DLs (20 and 40 mg). An overview of the incidence of the most frequent AEs per MedDRA SOC and PT regardless of relationship to study medication is provided in Table 2.

**TABLE 2:** Incidence of All AEs Occurring in ≥ 10% of Patients (MedDRA SOC and PT).

MedDRA SOC	MedDRA PT	*n*	%
Total number of patients		25	100.0
Number of patients with at least one AE		24	96.0
Blood and lymphatic system disorder		17	68.0
	Thrombocytopenia	11	44.0
	Neutropenia	5	20.0
	Anemia	4	16.0
	Febrile neutropenia	3	12.0
Gastrointestinal disorders		16	64.0
	Diarrhea	8	32.0
	Nausea	6	24.0
	Constipation	4	16.0
	Dry mouth	3	12.0
General disorders and administration site conditions		15	60.0
	Fatigue	5	20.0
	Edema peripheral	4	16.0
	Pyrexia	4	16.0
	General physical health deterioration	3	12.0
	Mucosal inflammation	3	12.0
Infections and infestations		15	60.0
	Pneumonia	4	16.0
Investigations		10	40.0
	C‐reactive protein increased	4	16.0
Nervous system disorders		10	40.0
	Dizziness	5	20.0
Respiratory thoracic and mediastinal disorders		9	36.0
	Epistaxis	6	24.0
	Dyspnea	4	16.0
Injury, poisoning, and procedural complications		7	28.0
	Fall	3	12.0
	Skin laceration	3	12.0
Vascular disorders		6	24.0
	Hypotension	4	16.0
Psychiatric disorders		6	24.0
	Confusional state	3	12.0
Skin and subcutaneous tissue disorders		6	24.0
Musculoskeletal and connective tissue disorders		5	20.0
	Arthralgia	3	12.0
Metabolism and nutrition disorders		4	16.0
Renal and urinary disorders		3	12.0

*Note*: Multiple mentions possible.

Abbreviations: AE, adverse events; MedDRA, Medical Dictionary for Regulatory Activities; PT, preferred term; SOC, system organ class.

The most frequently reported severe AEs (≥ grade 3) were thrombocytopenia (44.0% of patients), neutropenia (20.0%), anemia (16.0%), pneumonia (16.0%), febrile neutropenia (12.0%), and dyspnea (12.0%). Importantly, neurological, skin, and gastrointestinal toxicities were infrequent and of lower grade.

In total, five patients (20.0%) experienced an SAE leading to death; all of them were related to infections. There were no SUSAR (Suspected Unexpected Serious Adverse Reactions). No differentiation syndrome or unexpected interactions between TCP, LDAC, and ATRA were observed. A complete classification of all AEs and SAEs can be found in the Supplement (Tables S1 and S2).

### 3.5 Upregulation of CD38 Protein Expression on Bone Marrow Cells

**FIGURE 2:**
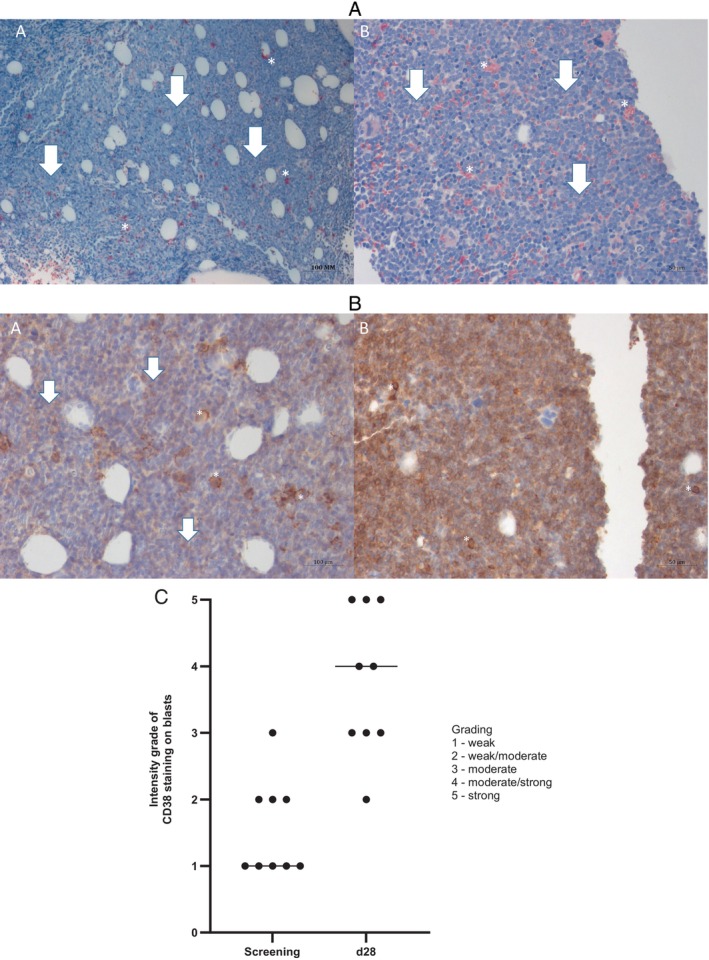
In vivo induction of CD38 protein expression on AML blasts. The images depict formalin‐fixed, paraffin‐embedded bone marrow trephine biopsy samples obtained from patient 01‐013 (DL 4). (A) The picture shows the NACE (Naphthol‐AS‐D Chloroacetate‐esterase) labeling of formalin‐fixed paraffin embedded bone marrow trephine biopsy samples (magnification 40X). Panel A represents the pretreatment sample, while panel B shows the post‐treatment (after 1 cycle on day 28) sample. There are repressed granulopoiesis (marked with white asterisks) and massive blast cell infiltration (> 90%) (marked with white arrows) on both panels. (B) The picture shows the CD38 labeling of formalin‐fixed paraffin embedded bone marrow trephine biopsy samples (magnification 40X). Panel A represents the pretreatment sample, while panel B shows the post‐treatment (after one cycle on day 28) sample. On panel A, low CD38 expression on the blast cells (marked with white arrows) is seen, while panel B shows strong expression of CD38 on the blast cells. As an internal positive control, plasma cells have been used (marked with white asterisks). (C) IHC staining intensity in bone marrow. Immunohistochemical analysis of bone marrow biopsies, collected from nine patients both pretreatment and postfirst cycle of therapy. Staining intensity categorized into 5 groups: weak—1, weak/moderate—2, moderate—3, moderate/strong—4, and strong—5.

Bone marrow biopsy samples from nine patients were available for analysis of immunohistological changes during the first cycle. One further pretreatment sample had to be excluded from the analysis due to poor preservation of the morphology. The biopsies were stained for differentiating neutrophils (NACE) and for expression of CD38 (Figure 2A,B) before and after the first cycle. All evaluable patients showed an increase in CD38 expression on marrow blasts after the first cycle. The median staining score increased from weak to moderate/strong (Figure 2C).

### 3.6 Changes in Fetal Hemoglobin Levels During Treatment

Fifteen patients were available for HbF analysis both before and after the first cycle. We observed a decreased fraction of HbF in peripheral blood after the first cycle compared to pretreatment levels (Figure S3). Median HbF% decreased significantly from 1.0% (range: 0.1%–4.3%) to 0.4% (range: 0.0%–1.0%) (*z* = −105.0, *p* = 0.0001), in line with persistent erythrocyte transfusion dependence in these patients. For patient 05‐003, who attained a PR, hemoglobin F increased to a maximum level of 2.9% after the seventh cycle. For patient 01‐005, who also attained a PR, hemoglobin F increased to a maximum of 1.9% after the fourth cycle. This patient also became transfusion‐independent for a total of 4 months following the second cycle. For patient 01‐005, median hemoglobin levels increased from 8.5 to 9.7 g/dL during the transfusion‐independent period.

### 3.7 TCP Plasma Levels

**FIGURE 3:**
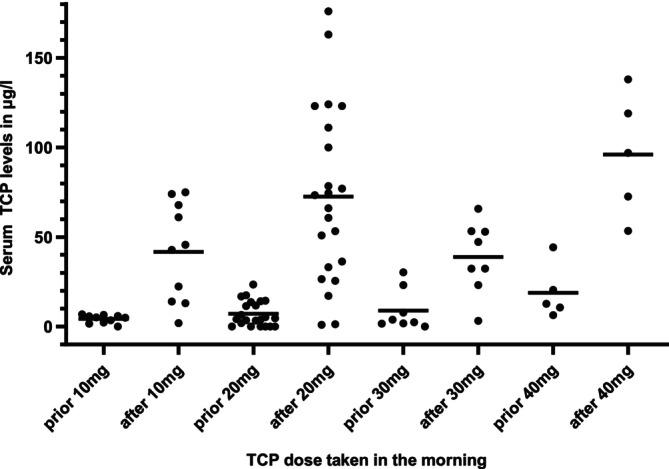
TCP plasma levels. TCP dose taken in the morning with patients' serum levels prior and two hours after intake with medians. Levels were measured at days 10, 20, 28, and 56. TCP was taken in the morning and evening as per protocol. Serum levels of TCP were quantified using a validated high‐performance liquid chromatography (HPLC) method coupled with tandem mass spectrometry (MS/MS).

Median TCP plasma trough levels and at 2 h after intake showed an increasing trend with higher TCP doses (Figure 3). Specifically, median TCP levels before intake were 5.05 μg/L (10 mg), 4.43 μg/L (20 mg), 3.12 μg/L (30 mg), and 12.7 μg/L (40 mg) (*F*_(3,9.631)_ = 2.233, *p* = 0.1494). Median TCP levels after intake were 44.25 μg/L (10 mg), 69.75 μg/L (20 mg), 39.80 μg/L (30 mg), and 96.90 μg/L (40 mg) (*F*_(3,25.66)_ = 5.224, *p* = 0.006).

### 3.8 Health‐Related Quality of Life, Depression and Anxiety

Data on quality of life were available for 16 patients. Median global health status at screening was 62.5 points (range: 16.7–91.7) and 41.7 points (range: 16.7–75.0) after the first cycle (Table S4). Thus, global health status measured by EORTC QLQ‐C30 showed a tendency toward worsening after the first cycle.

Data on psychological distress were available for 14 patients. The descriptive analysis of the anxiety sum score, measured by HADS‐D, indicated a worsening of the symptoms. However, median values of 4.0 points at baseline and 6.0 points after the first cycle can be interpreted as normal (Table S5). There were no patients with an abnormal scale. The number of patients with a borderline abnormal score increased from 2 at baseline to 5 after the first cycle.

There was a slight worsening of depressive symptoms, measured by HADS‐D, with median values of the sum score of 5.5 points at baseline and 8.0 points after the first cycle (Table S6). The number of patients with an abnormal score increased from 2 at baseline to 4 after the first cycle.

### 3.9 Overall Survival and Hematologic Responses

All 25 patients enrolled died during the study. The median overall survival time was 62 days (range: 14–325 days) (Figure 1B).

Results of the central response assessment were available for 22 patients (Table S7). Two of 25 patients (01‐005, 05‐003) achieved a PR (with temporary transfusion independence), both after the second cycle, and remained on trial for seven cycles. Myeloid panel sequencing revealed an *NPM1* and *IDH1* mutation in both patients. Twelve of 22 (54.5%) patients achieved at least SD. One patient (01‐009) had a reduction of bone marrow blasts from 79% to 15%, without completely fulfilling criteria for a PR, after the first cycle. The patient remained on trial for two cycles. NGS profiling showed, among others, a mutation in *NPM1*.

None of the five patients with mutations in *TP53* responded to treatment (four PD, one early death), and only three of nine patients with adverse cytogenetics achieved SD as best response.

### 3.10 In Vivo Transcriptional Changes Over Time in Peripheral Blood AML Blasts

Isolation and purification of peripheral blood (pb) blast over time during ongoing treatment was feasible for pretreatment and post‐treatment pb blasts in three patients (four on treatment timepoints in two, one on‐treatment timepoint in one patient, respectively). Global transcriptomic analysis was able to identify 29 (13 upregulated and 16 downregulated) DEGs (FDR < 0.05, Log2FC > 0.6) (Figure S4B,C). Limiting the stringency to *p* value < 0.05 (not adjusted *p* value) and Log2FC > 0.6 allowed us identification of 638 upregulated and 364 downregulated genes (Figure S4A,E,F). Those identified genes were able to segregate treated samples from untreated samples. On GSEA enrichment, we found depletion of TNF alpha signaling via NFkB with enrichment of proliferation‐associated pathways (E2F targets, G2M checkpoints) (Figure S4D).

**FIGURE 4:**
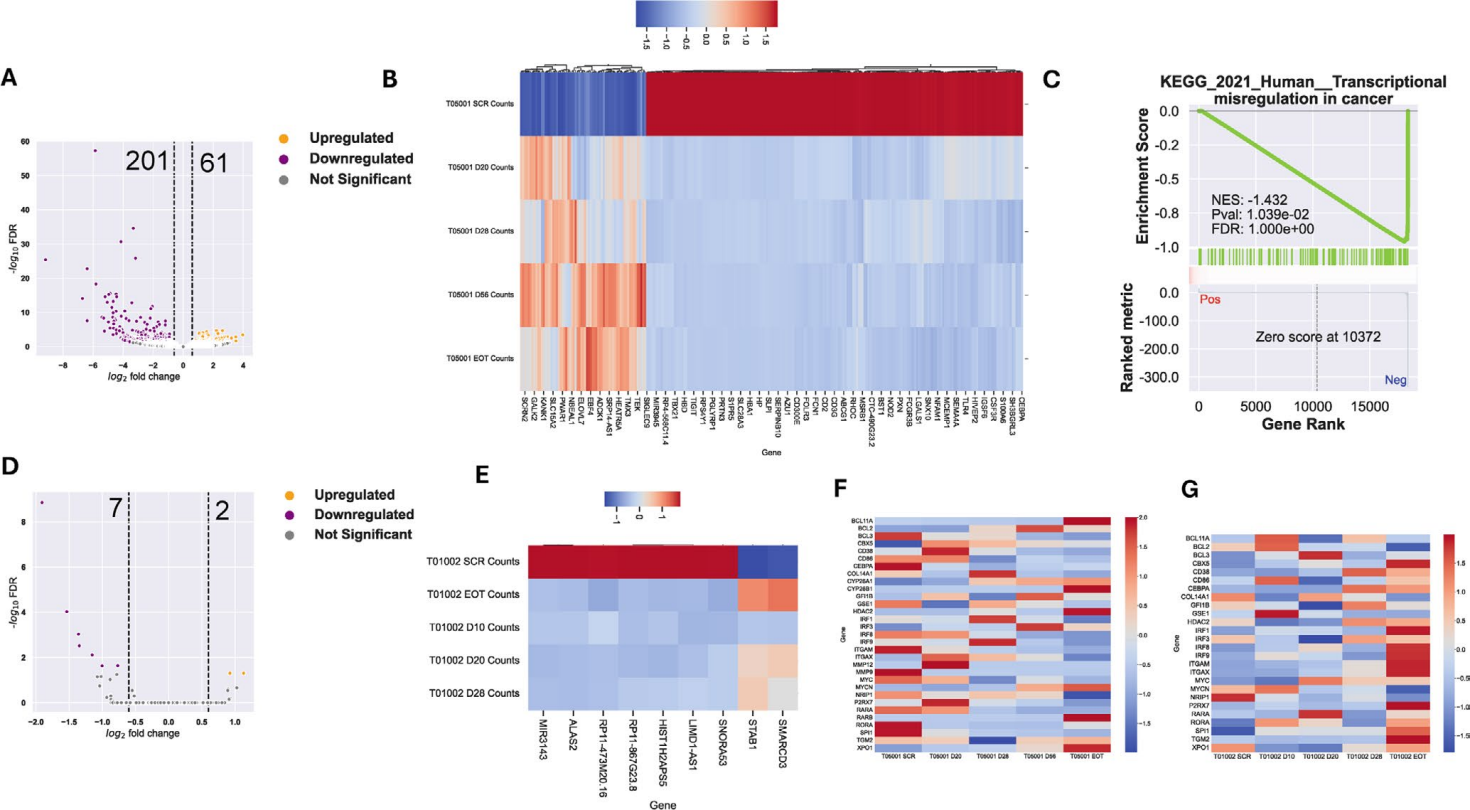
Treatment‐induced in vivo changes in global transcriptomics (serially purified peripheral blood AML blasts) (A, B) Volcano plot and Heatmap of differentially expressed genes (DEGs) (FDR < 0.05 & Log2foldchange > 0.6) in patient 05‐001 (dose level 2, treated for 78 days). (C) GSEA enrichment of transcriptome on ‘KEGG 2021 Human’ database. (D, E) Volcano plot and Heatmap of DEGs (FDR < 0.05 & Log2foldchange > 0.6) in patient 01‐002 (dose level 1, treated for 50 days). (F, G) Regulation of known LSD1‐regulated genes in patient 05‐001 (F) and patient 01‐002 (G).

We investigated patient 05‐001 (with four serial samples), having OS > 6 months and the 3rd longest exposure to DL 2 (40 mg/day) of TCP (78 days), and patient 01‐002 (52 days of exposure to DL 1 of 20 mg/day TCP) with survival of 54 days. Interestingly, we were able to identify 262 DEGs (61 up, 201 down) in treated samples compared to screening timepoint in patient 05‐001 (Figure 4A,B). Furthermore, upon GSEA on KEGG, we were able to identify depletion of ‘Transcriptional misregulation in cancer’ (Figure 4C) (Figure S5A) pointing toward the possibility that the TCP + ATRA regimen, at these specific doses, targets cancer associated perturbed transcription. In comparison, blasts from patient 01‐002 showed less transcriptional effects of treatment (Figure S5B).

To find the common effects of treatment on both patients, we next interrogated 31 genes known to be regulated by LSD1 inhibition (pharmacologically or by knock‐down) or ATRA treatment. For patient 05‐001, partially overlapping gene expression patterns and kinetics of regulation were noted: upregulation of *MMP12, CD38, P2RX7*, and *ITGAX* (day 20), *COL14A1, IRF1, IRF9* (day 28), *IRF3, GFI1B, BCL2* (day 56) and *HDAC2, BCL11A, CYP26B1, RARß*, and *XPO1* (day 76, EOT) (Figure 4F). As shown in Figure 4G, the majority of genes interrogated in blasts from patient 01‐002 was not found to be overexpressed prior to treatment, but sharply induced with treatment at least one on‐treatment timepoint, particularly at day 52 (EOT), highlighting a delayed effect due to level one dose. These induced genes include *ITGAM*, *ITGAX, P2RX7, TGM2, SPI.1/PU.1, CBX7, IRF1, IRF7*, and *IRF9*. Other upregulated genes included *GSE1, BCL2, BCL11A, CD86*, and *MYCN* (day 10), *RARA, BCL3*, and *C‐MYC* (day 20), and *CD38, C/EBP‐alpha, GFI1B, HDAC2*, and *IRF3* (day 28). In contrast, *NRIP1*, the gene with the highest expression prior to treatment start, was downregulated with continued treatment.

## 4 Discussion

Schenk and colleagues have demonstrated that pharmacological LSD1 inhibition in conjunction with ATRA provides a rational therapeutic approach in AML [8]. Others and ourselves have translated this approach clinically by employing TCP in combination with ATRA in patients with relapsed/refractory AML/MDS [21, 22]. Patients accrued to our study were mostly older (median age 75), often with an adverse genetic profile, and were heavily pretreated (92% refractory to prior HMA‐based treatment). Treatment was overall well tolerated on all DLs. The median duration of TCP exposure was 39.5 days, with several patients remaining on treatment for more than 3 months. This corresponds to a median of 1.4 cycles (range: 0.4–8.1), similar to the findings of Wass et al. (median 1.4 cycles, range: 0.5–3.5) [21]. Tayari et al. reported a median duration on study for 2.8 months (range: 0.4–9.2) [22]. While no complete and only two partial remissions were obtained, 45.5% of the evaluable patients achieved SD (comparable to the other studies), whereas no clinically relevant differentiation syndrome was observed in our trial.

In the study by Tayari et al., cutaneous side effects of ATRA were observed, leading to discontinuation in three patients. We hypothesize that the lack of severe ATRA‐related side effects in our study is also due to the scheduled interruption of ATRA at the beginning of every fourth cycle.

Considering that the majority of patients were older than 70 years of age, the feasibility of TCP dosing beyond day 28 was encouraging, even at the highest DL, and in line with published results on high‐dose TCP in treatment‐resistant depression [23]. Importantly, the observed side effects plateaued. Thus, 7 of 25 patients received TCP beyond day 56, three of them on the highest DL. Two patients (DL 2, 4) continued beyond 6 months of TCP (Figure 1A).

The measured TCP serum levels were similar to those measured by Wass et al. TCP levels had a tendency to be higher with increased doses, at trough and peak, suggesting accumulation and the development of a steady state.

This study showed a tendency toward worsening in both global health status and psychological distress after the first cycle of treatment. This decline was likely influenced by the increasing burden of the disease, which TCP was unable to fully mitigate. Future studies could benefit from using a questionnaire at baseline and after, for example, 3 or 6 months to assess the longer‐term impact of treatment on quality of life and psychological well‐being.

The majority of patients with adverse genetics, including *TP53* mutations, did not respond to treatment. The two patients attaining a PR exhibited mutations in *NPM1* and *IDH1*.

Concordant with the lack of erythroid responses, hemoglobin F levels were reduced at day 28. In the two PR patients, hemoglobin F increased after bone marrow blast reduction (delayed response in HbF levels likely reflects the time needed for recovery of erythropoiesis). Notably, we consistently observed the induction of CD38 on bone marrow blasts after the first cycle, which was in line with CD38 as a well‐established transcriptional target of ATRA. To the best of our knowledge, this is the first demonstration, in a prospective AML trial, of in vivo CD38 induction by an ATRA‐based treatment. With the availability of daratumumab and other anti‐CD38 antibodies, epigenetic priming to upregulate CD38 before antibody treatment has been studied in multiple myeloma and is also pursued in AML treatment by several groups, such as Buteyn et al. and Du et al. [24, 25].

Analysis of gene expression in two patients (05‐001 and 01‐002) revealed intriguing insights into the treatment's effects. Patient 05‐001, who received the higher TCP dose for a longer treatment period, exhibited distinct changes in gene expression, suggesting that TCP + ATRA effectively targets cancer‐related transcriptional pathways. While these changes were less pronounced in patient 01‐002 (who received a lower TCP dose for a shorter treatment period), both patients displayed overlapping gene expression patterns, particularly among genes regulated by LSD1. Notably, patient 01‐002 experienced a delayed response in the expression of these LSD1‐related genes. The above‐mentioned findings suggest that TCP + ATRA may exert its antileukemic effects by modulating specific transcriptional pathways, including those involving LSD1, and that dosage might play a crucial role in the timing and magnitude of these effects.

The most prominent effect is a shift toward a more differentiated myeloid phenotype, evidenced by the upregulation of key myeloid markers like SPI1, ITGAM, ITGAX, C/EBP‐alpha, and GFI1B [26]. Downregulation of the transcriptional corepressor NRIP1, which was highly expressed at baseline and has been linked to unresponsiveness to ATRA, may contribute to overcoming the leukemic differentiation block by facilitating a global increase in gene expression [27]. Concurrently, we observed changes suggesting epigenetic reprogramming, including alterations in CBX7 and HDAC2 expression, reflecting the possible direct impact of LSD1 inhibition on chromatin structure [28, 29]. Finally, the induction of TGM2 and alterations in BCL family members suggest that apoptosis may contribute, alongside differentiation, to the overall therapeutic effect.

It should be noted that, besides its histone demethylating function, LSD1 has also a scaffolding function. Inhibition of both is crucial in the treatment of AML [30, 31].

There are several limitations to our study. The observed antileukemic effect, even of repetitive cycles, did not result in documented complete remissions. While the gradual up‐dosing approach and combination with LDAC allowed us to achieve higher drug doses than in the other reported trials of TCP and ATRA, the measured trough levels remained below the levels required to observe a significant differentiation effect of TCP on leukemic cells in vitro [22, 30]. Hence, more potent and specific LSD1 inhibitors such as iadademstat and bomedemstat hold promise in AML and myeloproliferative syndromes [32, 33]. Due to the limited sample size, only a small number of patients could be included in the serial RNA analysis. The trial's slow accrual rate in 2020/2021, during the highest DL, did not allow for expansion beyond three patients fully evaluable for safety; therefore, we cannot exclude that DLTs might have occurred in the 3+ patients in phase II. This reduction in accrual coincided with the availability of venetoclax‐based combination treatments of R/R AML. Preclinical data [34, 35] provide a rationale for exploring the combination of LSD1 inhibition with venetoclax‐based therapies.

In conclusion, the combination of TCP with ATRA and LDAC was well‐tolerated and resulted in disease stabilization in almost half the patients in this heavily pretreated cohort. Our results suggest a limited cross‐resistance with prior HMA treatment. The lack of clinically significant differentiation syndrome may be due to the implementation of LDAC. Therefore, this concept warrants further consideration when investigating more potent LSD1 inhibitors in combination with ATRA. An alternative priming approach to re‐establish ATRA sensitivity in non‐M3 AML patients, by the decitabine‐venetoclax combination, is presently addressed in the randomized, placebo‐controlled, phase III DECIDER‐2 trial (AMLSG 32‐21, DRKS00023646).

## Author Contributions

Michael Kruszewski extracted and analyzed data, curated data, wrote the original draft of the manuscript, interpreted results, updated reference lists, and created the final tables and figures. Claudia Schmoor was involved in conceptualization, project administration, performed statistical analyses, created tables and revised the manuscript. Tobias Berg screened, identified, and recruited eligible patients, obtained informed consent, and contributed to writing – review and editing of the manuscript. Usama‐Ur Rehman and Kevin Moschallski were involved in RNA‐seq investigation, data curation, formal analysis, and visualization. Marcus Schittenhelm, Katharina Götze, Andrea Kündgen, and Caroline Pabst identified and recruited eligible patients and obtained informed consent. Tobias Ma performed cell sampling, data curation and formal analyses. Anna Frey was involved in investigation, data curation, and formal analysis. Julia Stomper analyzed data, contributed to methodology and writing – review and editing of the manuscript. Dietmar Pfeifer was involved in investigation, data curation, and formal analysis. Eric Metzger was involved in investigation. Johannes Jung identified and recruited eligible patients and contributed to writing – review and editing of the manuscript. Johanna Thomas contributed to writing – review and editing. Gesine Bug identified and recruited eligible patients and obtained informed consent. Justus Duyster was involved in project conceptualization and provided resources. Manfred Jung was involved in project conceptualization and funding acquisition. Roland Schüle was involved in project conceptualization and funding acquisition. Ralph Wäsch contributed to conceptualization, methodology, and project administration. Olga Grishina was centrally involved in conceptualization, project administration, methodology, investigation, data curation, formal analysis and writing – review and editing of the manuscript. Michael Lübbert was responsible for conceptualization, funding acquisition, project administration, resources, supervision, methodology, and writing – review and editing of the manuscript.

## Ethics Statement

The trial protocol was approved by the ethics Committee of the University of Freiburg.

## Consent

Written informed consent was obtained according to international guidelines and local laws.

## Conflicts of Interest

The authors declare no conflicts of interest.

## Supporting information


**Data S1.** Supporting Information.

**Tables S1 and S2.** Supporting Information.

## Data Availability

The RNA sequencing data generated during this study has been deposited in the Gene Expression Omnibus (GEO) repository. These data can be accessed under accession number (GSE277031, reviewer token—uzcjocmortytxod). Additional data related to adverse events are available in the Supporting Informations.
