# Supplementary material for: Sensitization of Non‐M3 Acute Myeloid Leukemia Blasts to All‐Trans Retinoic Acid by the LSD1 Inhibitor Tranylcypromine: TRANSATRA Phase I Study
Source: Eur J Haematol. 2025 Jun 3;115(3):266–77. doi: 10.1111/ejh.14426 (PMC12319880; doi:10.1111/ejh.14426)
Supplement: Supplementary file 1 — Data S1. Supporting Information. [file EJH-115-266-s002.docx]

**Supplementary Figure 1** NGS mutational profiling of pretreatment blasts (CD34+ and/or CD117+) from 23 patients (Illumina Myeloid Panel, not available for patient 05-006, known JAK2 mutation, and patient 06-001). The following 73 genes were analysed: ABL1, ANKRD26, ASXL1, ASXL2, BCOR, BCORL1, BRAF, CALR, CBL, CEBPA, CD33, CCND1, CCND2, CSF3R, CSFR1, CSNK1A1, CUX1, DDX41, DNMT3A, ETV6, ETNK1, EZH2, FBXW7, FLT3, GATA1, GATA2, IDH1, IDH2, IKZF1, JAK1, JAK2, JAK3, KIT, KRAS, MPL, MYC, MYD88, NFE2, NF1, NOTCH2, NPM1, NRAS, NSD1, PIGA, PIK3CA, PHF6, PPM1D, PRPF8, PTPN11, RAD21, RUNX1, SETBP1, SETD2, SF3B1, SH2B3, SMC1A, SMC3, SRP72, SRSF2, STAT3, STAT5B, STAG2, SUZ12, TERC, TERT, TET2, TP53, U2AF1, UBA1, WT1, ZBTB33, ZBTB7A and ZRSR2. Intronic and silent variants, as well as known SNPs, were excluded from the analysis. The significance threshold was set at 3%.


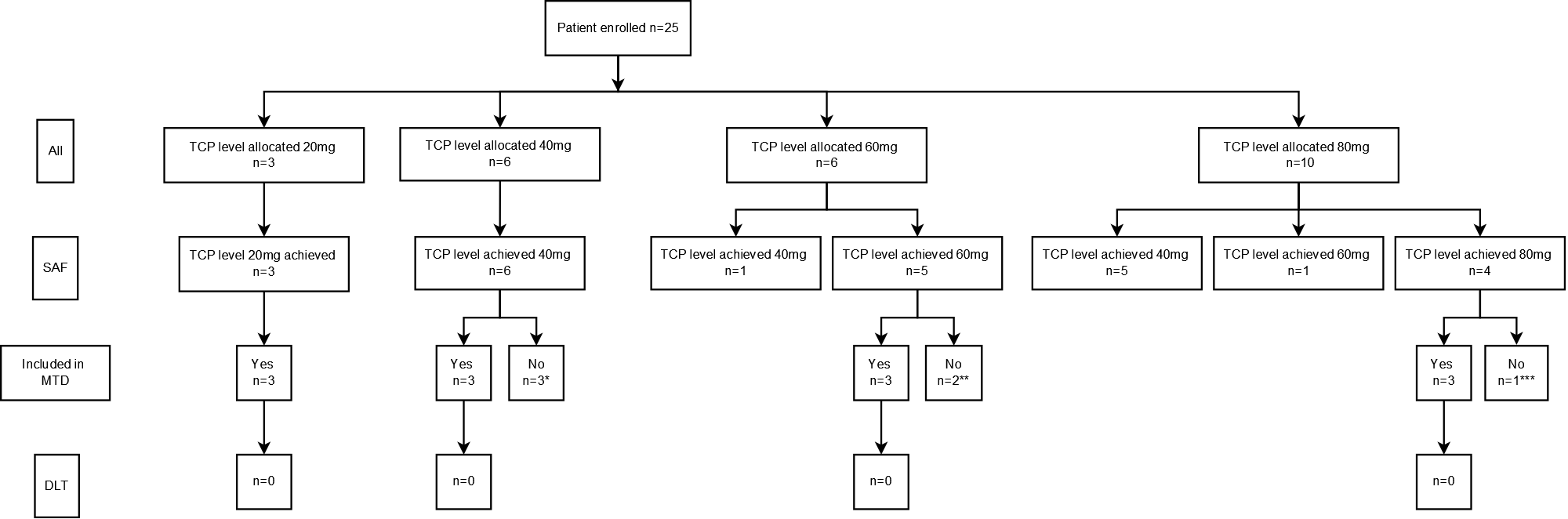


**Supplementary Figure 2** Consort diagram with number of patients per allocated and achieved dose level. *Reason: Drug exposure not sufficient (to AraC n=1, to ATRA n=2), **Reason: Drug exposure not sufficient (to ATRA n=1), Relapse (n=1), ***Reason: Drug exposure not sufficient (to ATRA n=1)


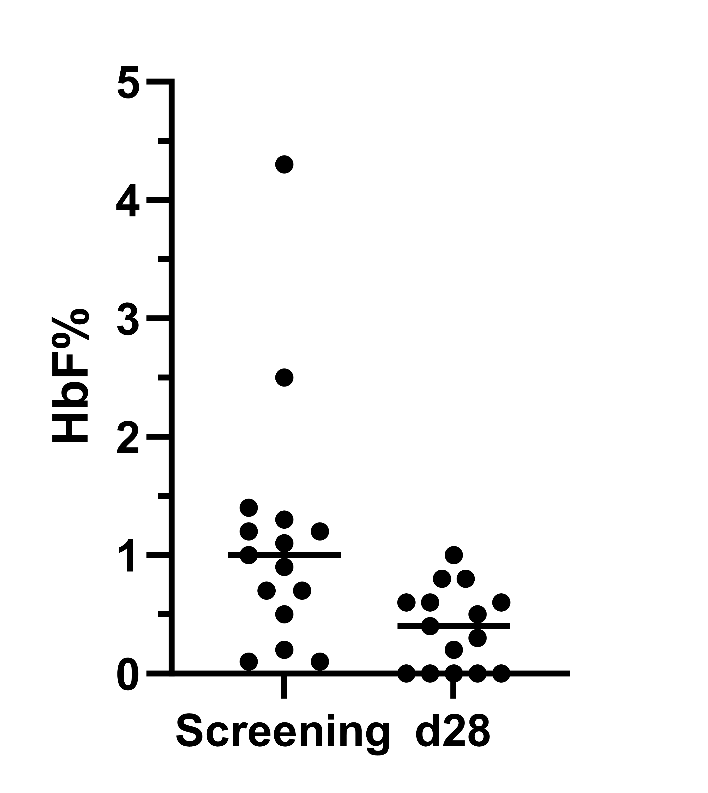


**Supplementary Figure 3** Fractions of fetal hemoglobin (HbF%) in peripheral blood before and after the first cycle (n=15).


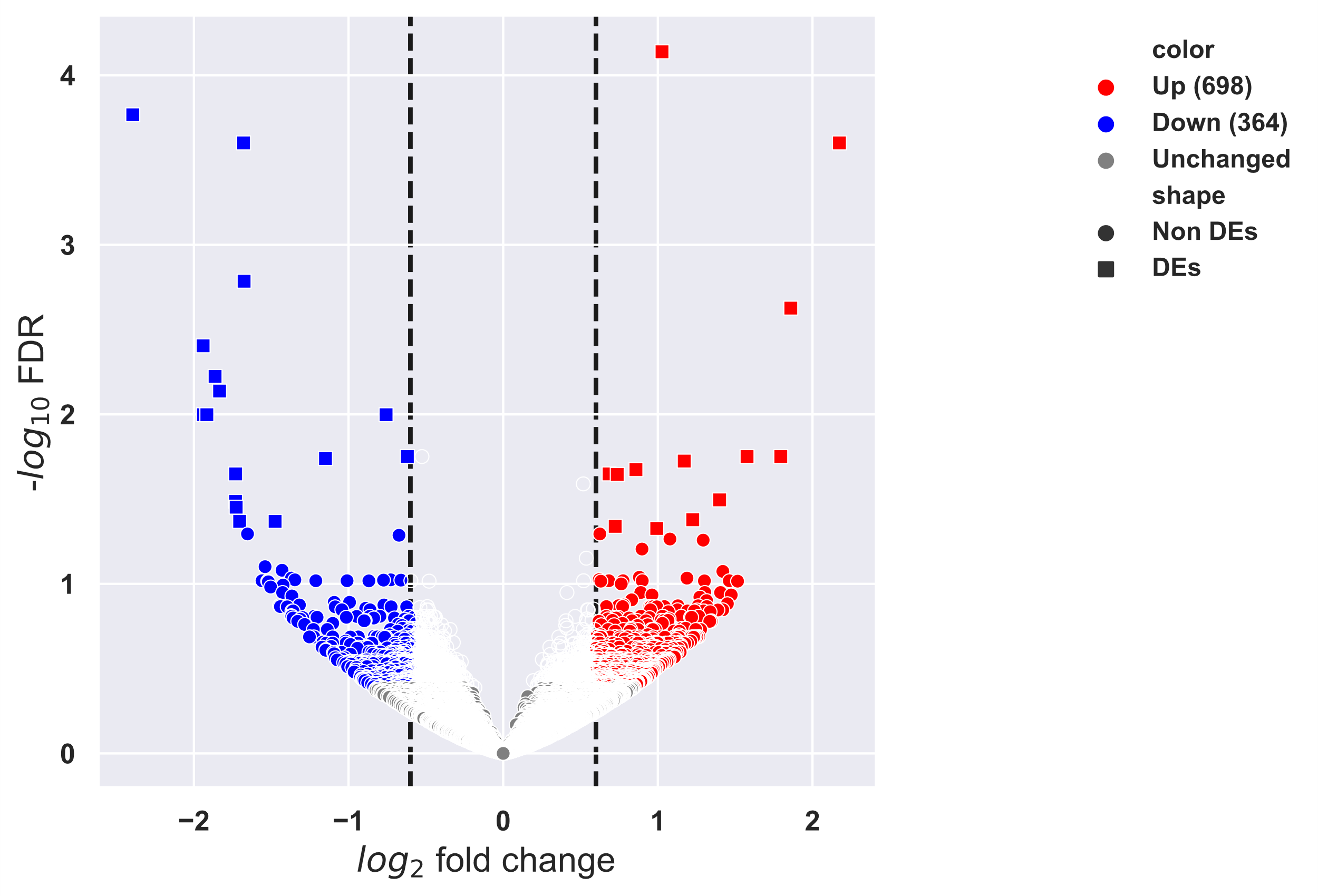

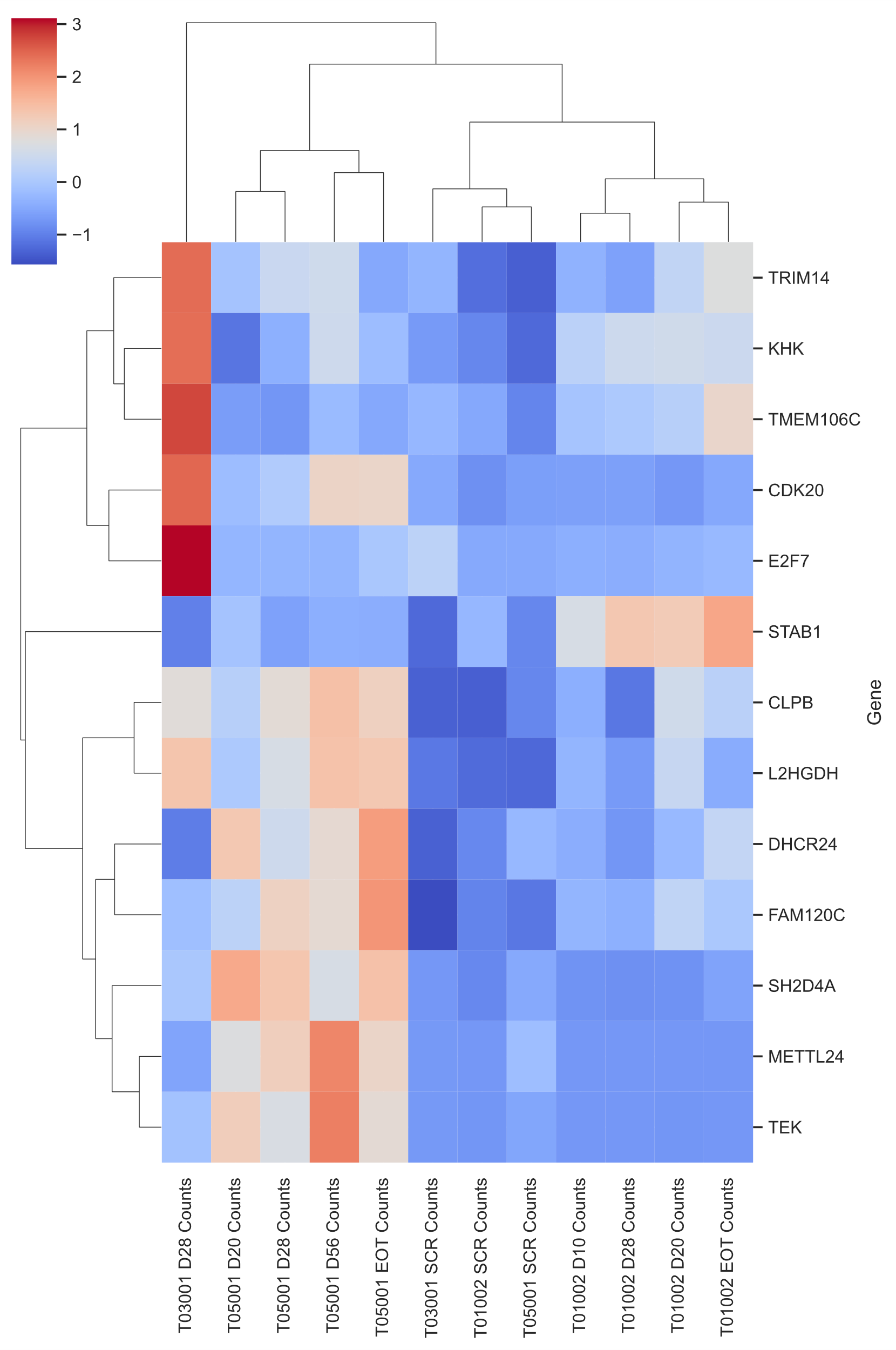

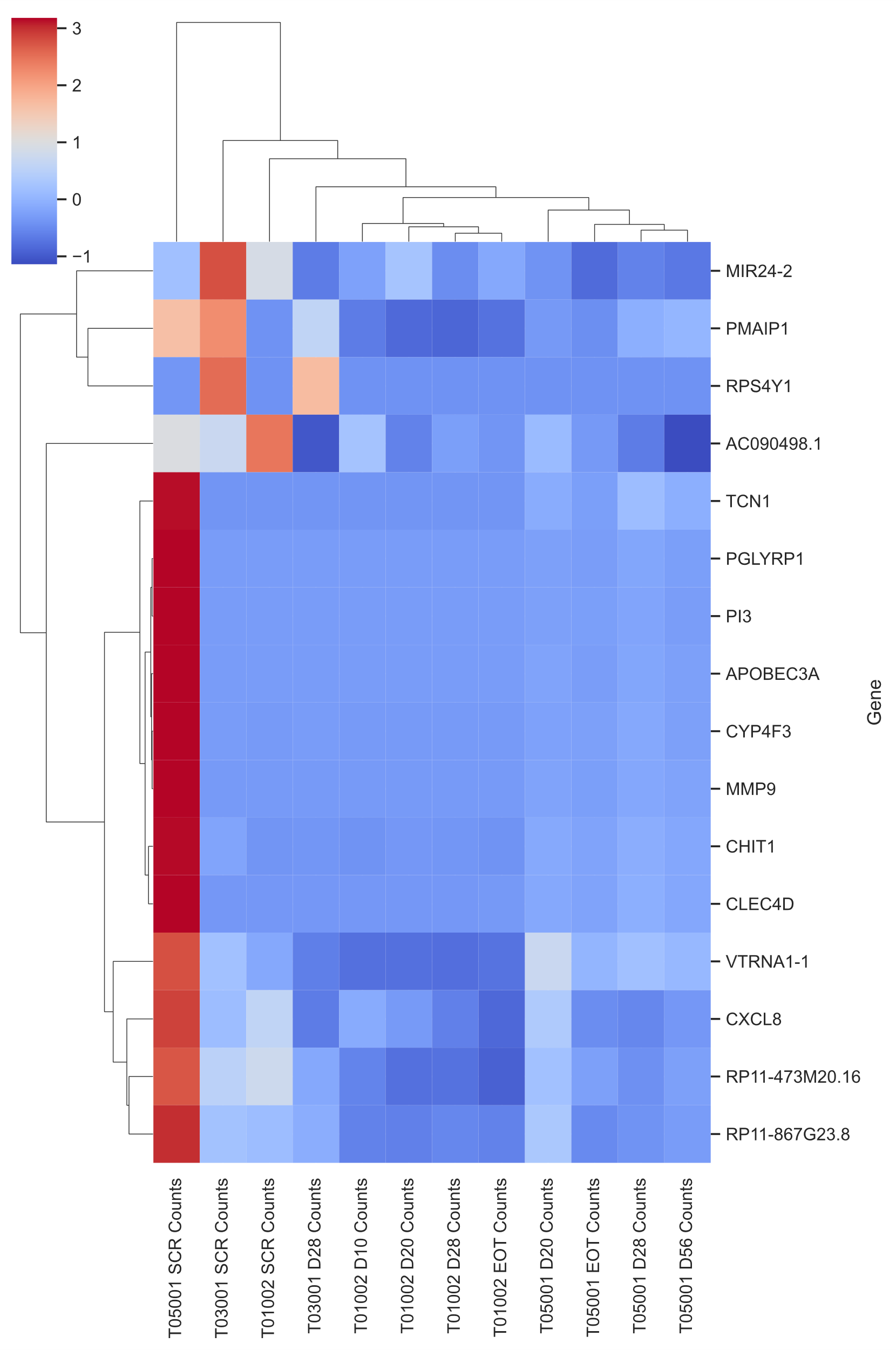


Downregulated DEGs: 16

Upregulated DEGs: 13

**C**

**B**

**A**


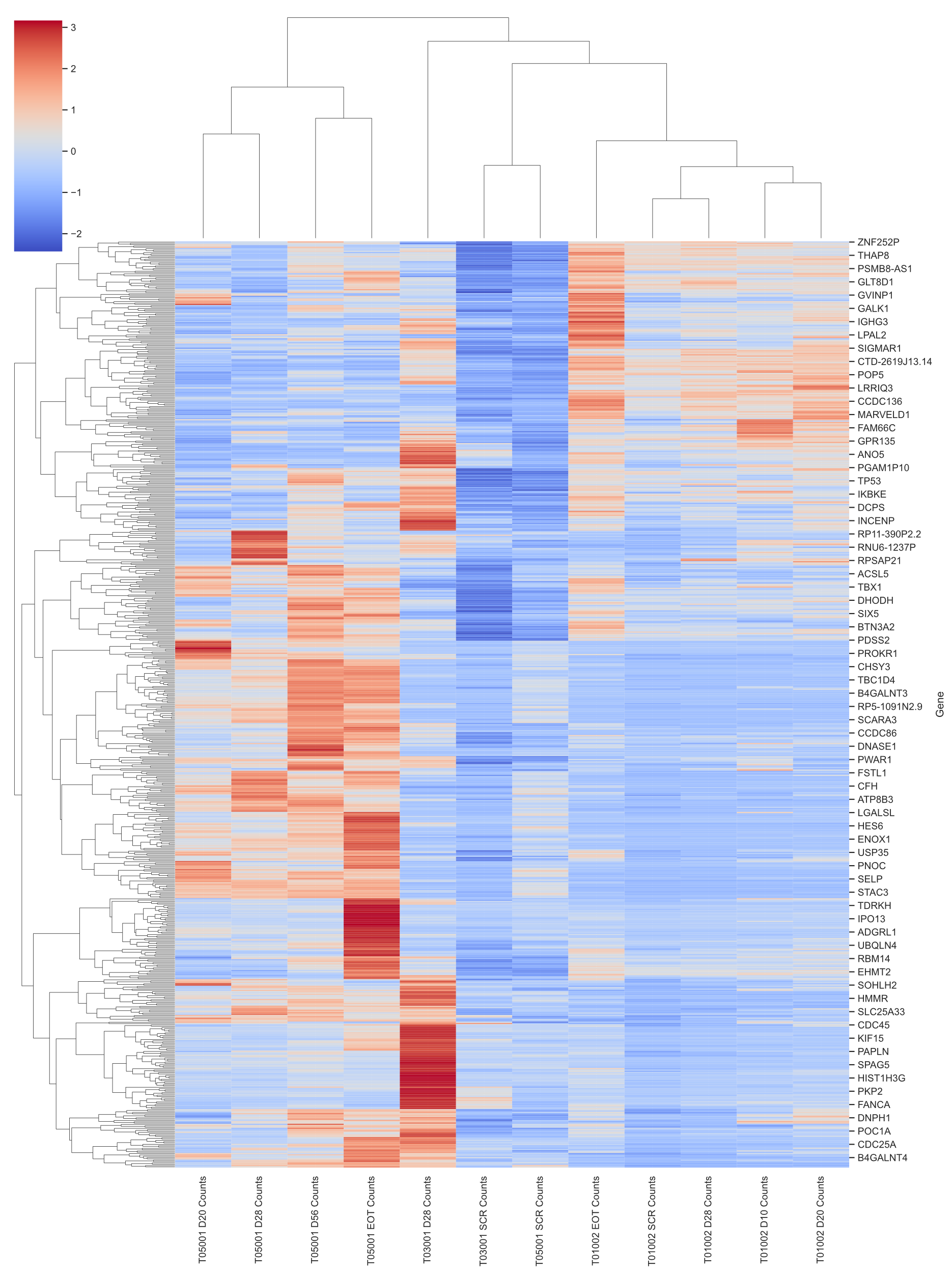


Upregulated: 638


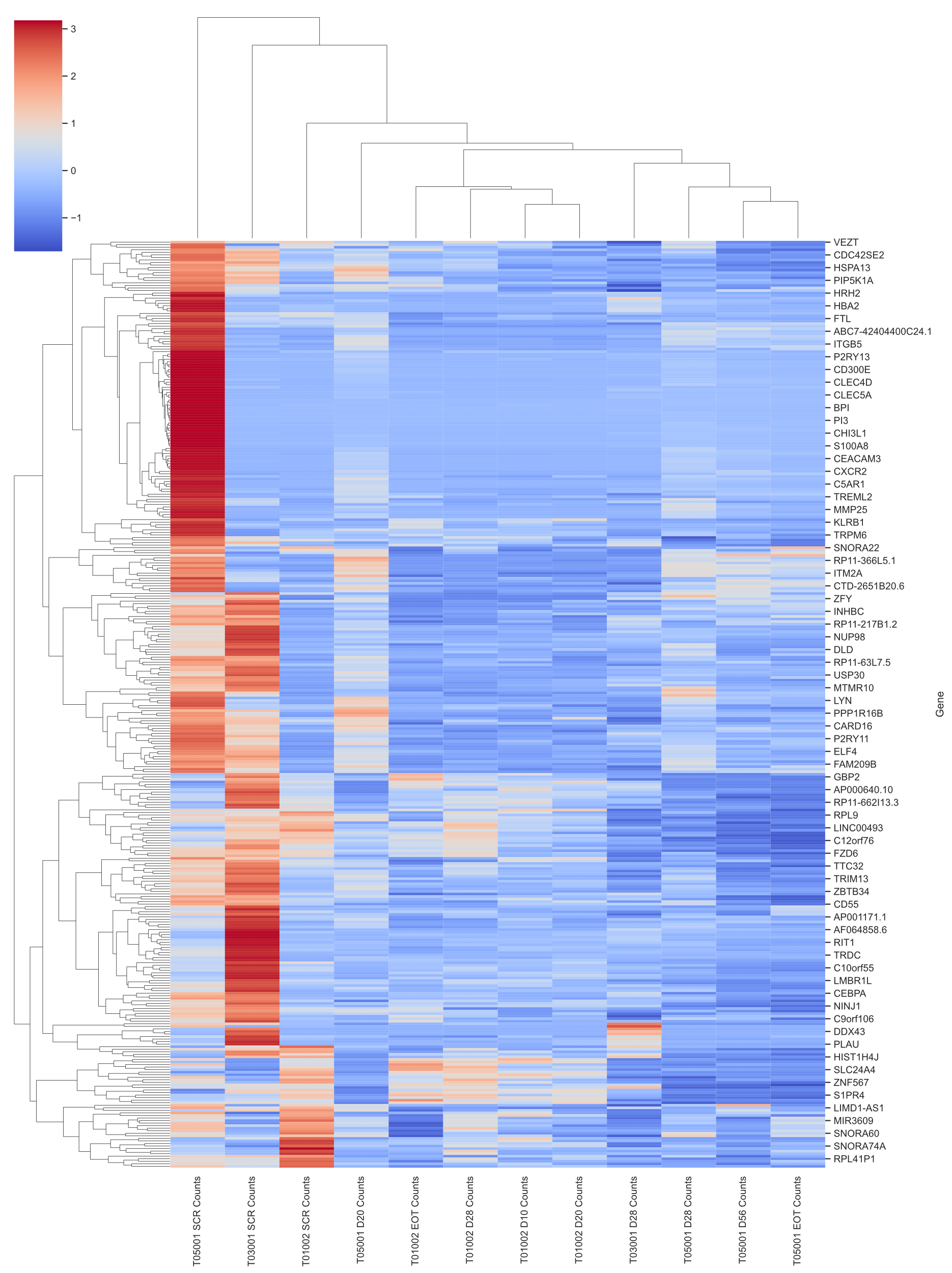


Downregulated: 364

**F**

**D**

**E**


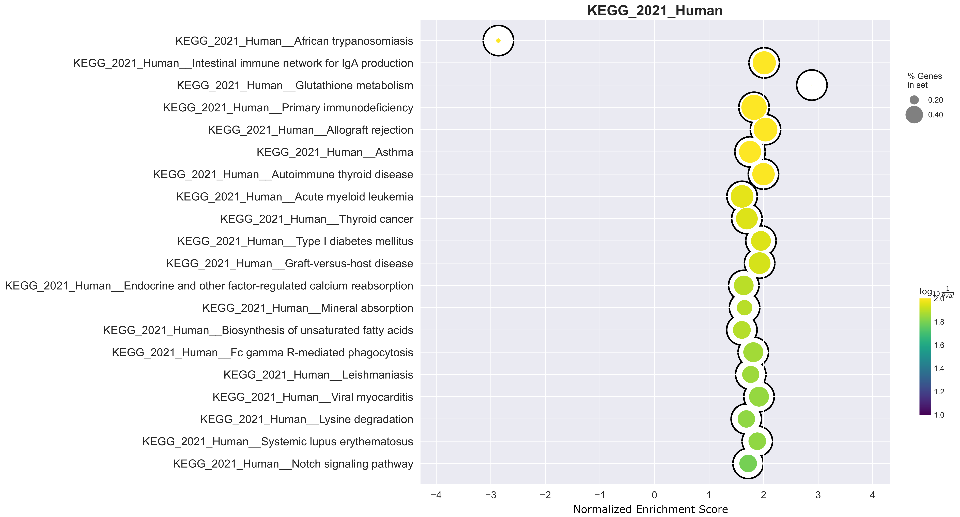


**Supplementary Figure 4 Treatment-induced *in vivo* changes in global transcriptomics (serially purified peripheral blood AML blasts from 3 patients)** For three patients, post-treatment start blast isolates were available: 4 from patient 01-002 (treated on dose level 1), 4 from patient 05-001 (treated on dose level 2), 1 from patient 03-001 (treated on dose level 3). (A) Volcano plot (B, C) heatmaps of upregulated and downregulated DEGs, respectively. (D) GSEA enrichment of transcriptome on the ‘Hallmark 50 pathways’ database, with cutoff of ‘FDR q value<0.1’. (E, F) Upregulated and downregulated genes, respectively, with a p value of <0.05 and Log2 fold change>0.6.

**
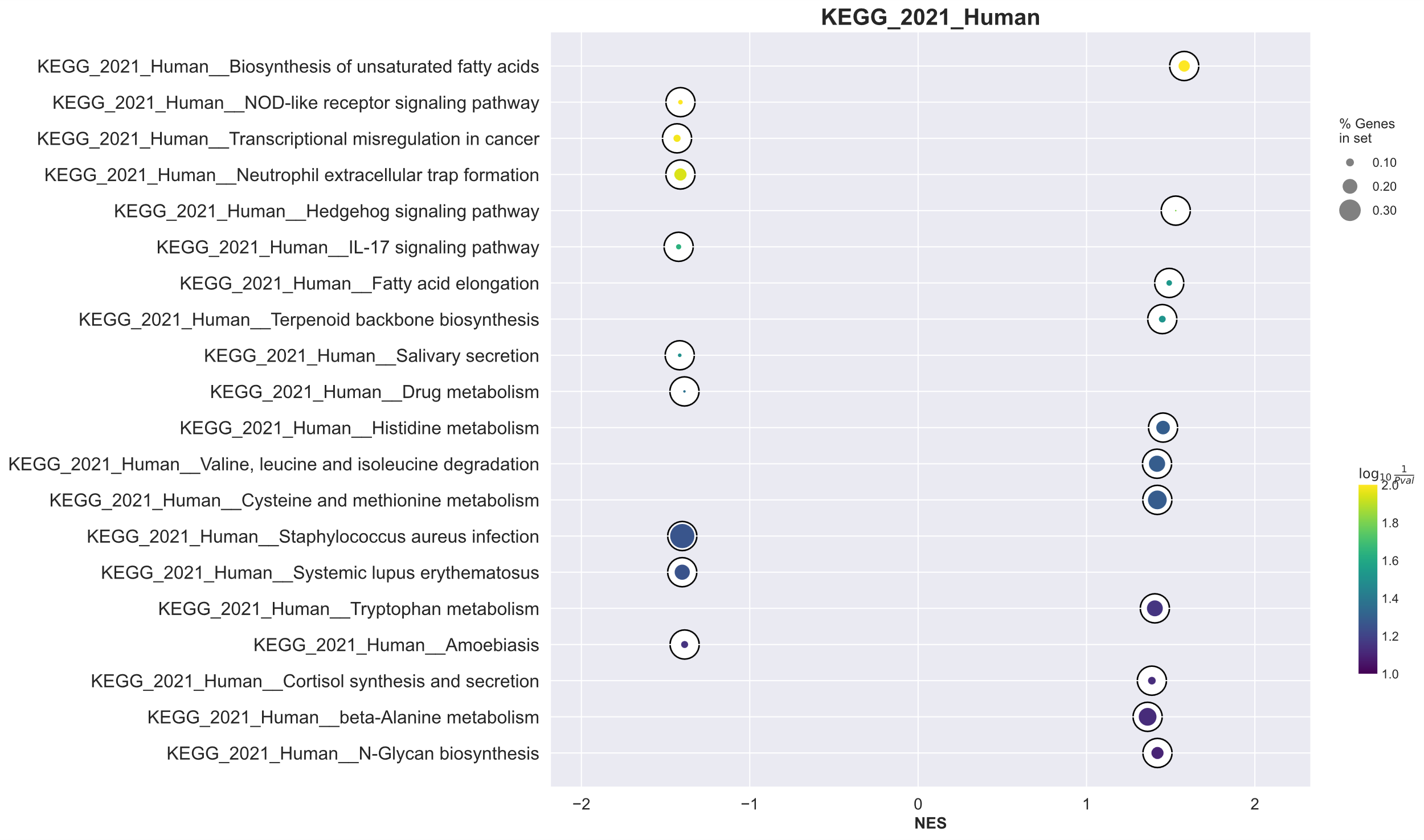

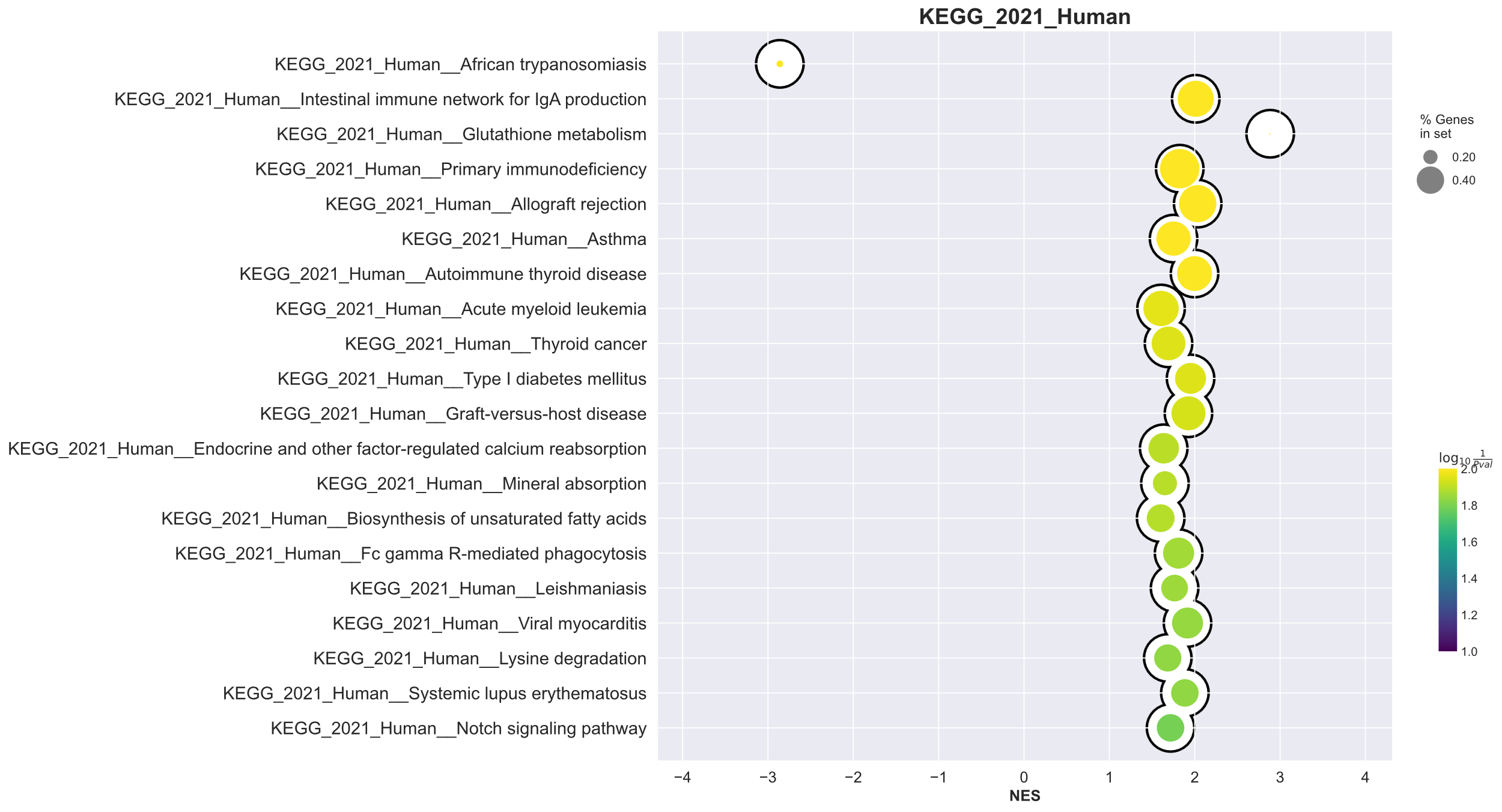
**

**B**

Patient 01-002

**A**

Patient 05-001

**Supplementary Figure 5 Differential transcriptomic enrichment in two serially studied patients.** (A) GSEA enrichment of patient 05-001 transcriptome on ‘KEGG 2021 Human’ database with cutoff of ‘NOM p value<0.1’ (B) GSEA enrichment of patient 01-002 transcriptome on ‘KEGG 2021 Human’ database with cutoff of ‘NOM p value<0.1’

.

**Supplementary Table 3**

| **Inclusion criteria** | 1. Patients ≥18 years (no upper age limit) 2. AML (WHO) or intermediate or higher risk MDS/CMML (IPSS-R >3.0); 3. No standard treatment available (comorbidities, higher age, refractoriness to standard or salvage chemotherapy and allografting, azanucleosides failure*); 4. Patients with <30,000 leukocytes/µl; 5. ECOG 0,1,2; 6. Written informed consent obtained according to international guidelines and local laws; 7. Ability to understand the nature of the trial and the trial-related procedures and to comply with them.   *Azanucleosides failure is defined as 1) no response after at least three (AML) or six (MDS) cycles of azacitidine or decitabine, 2) disease progression under treatment or 3) grade 3-4 non-hematologic toxicity. |
| --- | --- |
| **Exclusion criteria** | 1. Acute promyelocytic leukemia (APL, FAB M3); 2. Eligibility for standard induction or consolidation chemotherapy, immediate allografting, or a hypomethylating agent; 3. AML with CNS involvement; 4. AraC treatment within one month prior to registration; 5. Prior exposure to histone deacetylase inhibitors, including sodium valproate within one month prior to registration; 6. Stem cell transplant patient with GvHD or under systemic immunosuppression; 7. Previous gastrointestinal surgery that might interfere with drug absorption; 8. Pheochromocytoma; 9. Carcinoid tumor; 10. Confirmed or suspected cerebrovascular disease; 11. Vascular malformations including aneurysm; 12. Severe renal insufficiency; 13. Severe or poorly controlled hypertension; 14. Severe cardiovascular disease; 15. Hepatic insufficiency/liver disease; 16. Porphyria; 17. Diabetes insipidus; 18. History or presence of malignant hyperthermia; 19. Known psychiatric disorders; 20. Known allergy against soy beans or peanuts; 21. Known hypersensitivity to or intolerance of one of the trial drugs or its constituents (e.g., lactose, corn starch, indigocarmin (TCP), corn starch (AraC), other retinoids (ATRA)); 22. Simultaneous intake of the prohibited medication, incl. linezolid, that is likely to cause interactions (see detailed list section 6.6); 23. Patients who refuse to follow study-specific dietary guidelines; 24. Known or persistent abuse of medication, drugs, or alcohol; 25. Current or planned pregnancy, nursing period; 26. Failure to use safe methods of contraception; 27. Simultaneous participation in other interventional trials which could interfere with this trial and/or participation before the end of a required restriction period; 28. Participation in a clinical trial within the last 30 days before the start of this trial; 29. Persons who are in a relationship of dependence/employment with the sponsor or the investigator. |

**Supplementary Table 4** EORTC QLQ-C30 Scores

| **Visit [points]** | **Total*** | **Mean** | **STD** | **Min** | **Median** | **Max** |
| --- | --- | --- | --- | --- | --- | --- |
| Screening | 16 | 55.2 | 24.1 | 16.7 | 62.5 | 91.7 |
| After cycle 1 | 16 | 42.2 | 16.2 | 16.7 | 41.7 | 75.0 |

EORTC QLQ C30 scores at screening and after the first cycle. *Only patients with available data both at screening and after the first cycle were considered.

**Supplementary Table 5** HADS-D Anxiety sum score

| **Visit [points]** | **Total*** | **Mean** | **STD** | **Min** | **Median** | **Max** |
| --- | --- | --- | --- | --- | --- | --- |
| Screening | 14 | 3.86 | 3.01 | 0.0 | 4.0 | 8.0 |
| After cycle 1 | 14 | 5.08 | 3.78 | 0.0 | 6.0 | 10.0 |

HADS Anxiety sum scores at screening and after the first cycle. *Only patients with available data both at screening and after cycle 1 were considered.

**Supplementary Table 6** HADS-D Depression sum score

| **Visit [points]** | **Total*** | **Mean** | **STD** | **Min** | **Median** | **Max** |
| --- | --- | --- | --- | --- | --- | --- |
| Screening | 14 | 6.43 | 5.75 | 1.0 | 5.5 | 19.0 |
| After cycle 1 | 14 | 8.43 | 4.91 | 2.0 | 8.0 | 20.0 |

HADS Depression sum scores at screening and after the first cycle. * Only patients with available data both at screening and after cycle 1 were considered.

**Supplementary Table 7** Best response

|  | **n=22** |
| --- | --- |
| **Best response, n (%)** |  |
| Complete remission (CR) | 0 (0.0) |
| Partial remission (PR) | 2 (9.1) |
| Stable disease (SD) | 10 (45.5) |
| Progressive disease (PD) | 10 (45.5) |

Response according to Cheson et al. 2003 for AML and by Cheson et al. 2006 for MDS. Response evaluation was not possible in three patients. Three patients did not finish the first cycle due to clinical deterioration.
