# Supplementary material for: Sensitization of Non‐M3 Acute Myeloid Leukemia Blasts to All‐Trans Retinoic Acid by the LSD1 Inhibitor Tranylcypromine: TRANSATRA Phase I Study
Source: Eur J Haematol. 2025 Jun 3;115(3):266–77. doi: 10.1111/ejh.14426 (PMC12319880; doi:10.1111/ejh.14426)
Supplement: Supplementary file 2 — Tables S1 and S2. Supporting Information. [file EJH-115-266-s001.docx]

| **Supplementary table 1**: Incidence of AEs by System Organ Class (SOC), and Preferred Term (PT): All AEs |
| --- |

| *Supplementary table 1: Incidence of AEs by System Organ Class (SOC), and Preferred Term (PT): All AEs* | | | | | | | | | | | |
| --- | --- | --- | --- | --- | --- | --- | --- | --- | --- | --- | --- |
|  | | *Level achieved* | | | | | | | |  | |
|  | | *Level 1* | | *Level 2* | | *Level 3* | | *Level 4* | | *Total* | |
| *System organ class* | *Preferred term* | *No.* | *Total %* | *No.* | *Total %* | *No.* | *Total %* | *No.* | *Total %* | *No.* | *Total %* |
| Total number of patients |  | 3 | 100.0 | 12 | 100.0 | 6 | 100.0 | 4 | 100.0 | 25 | 100.0 |
| Number of patients with at least one AE |  | 3 | 100.0 | 11 | 91.7 | 6 | 100.0 | 4 | 100.0 | 24 | 96.0 |
| Blood and lymphatic system disorders |  | 3 | 100.0 | 4 | 33.3 | 6 | 100.0 | 4 | 100.0 | 17 | 68.0 |
|  | Thrombocytopenia | 3 | 100.0 | 3 | 25.0 | 3 | 50.0 | 2 | 50.0 | 11 | 44.0 |
|  | Neutropenia | 0 | 0.0 | 1 | 8.3 | 2 | 33.3 | 2 | 50.0 | 5 | 20.0 |
|  | Anaemia | 1 | 33.3 | 2 | 16.7 | 1 | 16.7 | 0 | 0.0 | 4 | 16.0 |
|  | Febrile neutropenia | 0 | 0.0 | 1 | 8.3 | 0 | 0.0 | 2 | 50.0 | 3 | 12.0 |
|  | Leukocytosis | 0 | 0.0 | 1 | 8.3 | 0 | 0.0 | 1 | 25.0 | 2 | 8.0 |
|  | Leukopenia | 0 | 0.0 | 0 | 0.0 | 1 | 16.7 | 0 | 0.0 | 1 | 4.0 |
|  | Pancytopenia | 0 | 0.0 | 0 | 0.0 | 1 | 16.7 | 0 | 0.0 | 1 | 4.0 |
| Gastrointestinal disorders |  | 2 | 66.7 | 8 | 66.7 | 3 | 50.0 | 3 | 75.0 | 16 | 64.0 |
|  | Diarrhoea | 1 | 33.3 | 3 | 25.0 | 2 | 33.3 | 2 | 50.0 | 8 | 32.0 |
|  | Nausea | 1 | 33.3 | 3 | 25.0 | 1 | 16.7 | 1 | 25.0 | 6 | 24.0 |
|  | Constipation | 0 | 0.0 | 3 | 25.0 | 0 | 0.0 | 1 | 25.0 | 4 | 16.0 |
|  | Dry mouth | 1 | 33.3 | 2 | 16.7 | 0 | 0.0 | 0 | 0.0 | 3 | 12.0 |
|  | Gastrointestinal haemorrhage | 0 | 0.0 | 0 | 0.0 | 1 | 16.7 | 1 | 25.0 | 2 | 8.0 |
|  | Anal incontinence | 1 | 33.3 | 0 | 0.0 | 0 | 0.0 | 0 | 0.0 | 1 | 4.0 |
|  | Dysphagia | 0 | 0.0 | 1 | 8.3 | 0 | 0.0 | 0 | 0.0 | 1 | 4.0 |
|  | Gastric haemorrhage | 0 | 0.0 | 0 | 0.0 | 0 | 0.0 | 1 | 25.0 | 1 | 4.0 |
|  | Haemorrhoids | 0 | 0.0 | 1 | 8.3 | 0 | 0.0 | 0 | 0.0 | 1 | 4.0 |
|  | Lower gastrointestinal haemorrhage | 0 | 0.0 | 0 | 0.0 | 1 | 16.7 | 0 | 0.0 | 1 | 4.0 |
|  | Melaena | 0 | 0.0 | 1 | 8.3 | 0 | 0.0 | 0 | 0.0 | 1 | 4.0 |
|  | Mouth haemorrhage | 0 | 0.0 | 1 | 8.3 | 0 | 0.0 | 0 | 0.0 | 1 | 4.0 |
|  | Oedema mouth | 0 | 0.0 | 0 | 0.0 | 0 | 0.0 | 1 | 25.0 | 1 | 4.0 |
|  | Oral mucosal discolouration | 0 | 0.0 | 1 | 8.3 | 0 | 0.0 | 0 | 0.0 | 1 | 4.0 |
|  | Vomiting | 0 | 0.0 | 0 | 0.0 | 1 | 16.7 | 0 | 0.0 | 1 | 4.0 |
| General disorders and administration site conditions |  | 2 | 66.7 | 7 | 58.3 | 4 | 66.7 | 2 | 50.0 | 15 | 60.0 |
|  | Fatigue | 1 | 33.3 | 3 | 25.0 | 1 | 16.7 | 0 | 0.0 | 5 | 20.0 |
|  | Oedema peripheral | 1 | 33.3 | 2 | 16.7 | 0 | 0.0 | 1 | 25.0 | 4 | 16.0 |
|  | Pyrexia | 1 | 33.3 | 2 | 16.7 | 0 | 0.0 | 1 | 25.0 | 4 | 16.0 |
|  | General physical health deterioration | 0 | 0.0 | 2 | 16.7 | 1 | 16.7 | 0 | 0.0 | 3 | 12.0 |
|  | Mucosal inflammation | 0 | 0.0 | 1 | 8.3 | 1 | 16.7 | 1 | 25.0 | 3 | 12.0 |
|  | Asthenia | 0 | 0.0 | 0 | 0.0 | 1 | 16.7 | 0 | 0.0 | 1 | 4.0 |
|  | Chest pain | 0 | 0.0 | 1 | 8.3 | 0 | 0.0 | 0 | 0.0 | 1 | 4.0 |
|  | Injection site haematoma | 0 | 0.0 | 1 | 8.3 | 0 | 0.0 | 0 | 0.0 | 1 | 4.0 |
|  | Peripheral swelling | 0 | 0.0 | 0 | 0.0 | 1 | 16.7 | 0 | 0.0 | 1 | 4.0 |
| Infections and infestations |  | 2 | 66.7 | 8 | 66.7 | 3 | 50.0 | 2 | 50.0 | 15 | 60.0 |
|  | Pneumonia | 0 | 0.0 | 3 | 25.0 | 0 | 0.0 | 1 | 25.0 | 4 | 16.0 |
|  | Sepsis | 1 | 33.3 | 1 | 8.3 | 0 | 0.0 | 0 | 0.0 | 2 | 8.0 |
|  | Abscess | 0 | 0.0 | 1 | 8.3 | 0 | 0.0 | 0 | 0.0 | 1 | 4.0 |
|  | Bacterial infection | 0 | 0.0 | 0 | 0.0 | 1 | 16.7 | 0 | 0.0 | 1 | 4.0 |
|  | Candida infection | 0 | 0.0 | 1 | 8.3 | 0 | 0.0 | 0 | 0.0 | 1 | 4.0 |
|  | Clostridium difficile colitis | 0 | 0.0 | 0 | 0.0 | 1 | 16.7 | 0 | 0.0 | 1 | 4.0 |
|  | Device related infection | 1 | 33.3 | 0 | 0.0 | 0 | 0.0 | 0 | 0.0 | 1 | 4.0 |
|  | Diarrhoea infectious | 0 | 0.0 | 1 | 8.3 | 0 | 0.0 | 0 | 0.0 | 1 | 4.0 |
|  | Escherichia infection | 0 | 0.0 | 1 | 8.3 | 0 | 0.0 | 0 | 0.0 | 1 | 4.0 |
|  | Escherichia sepsis | 0 | 0.0 | 1 | 8.3 | 0 | 0.0 | 0 | 0.0 | 1 | 4.0 |
|  | Haematoma infection | 0 | 0.0 | 1 | 8.3 | 0 | 0.0 | 0 | 0.0 | 1 | 4.0 |
|  | Medical device site infection | 0 | 0.0 | 0 | 0.0 | 0 | 0.0 | 1 | 25.0 | 1 | 4.0 |
|  | Neutropenic infection | 0 | 0.0 | 0 | 0.0 | 0 | 0.0 | 1 | 25.0 | 1 | 4.0 |
|  | Neutropenic sepsis | 0 | 0.0 | 1 | 8.3 | 0 | 0.0 | 0 | 0.0 | 1 | 4.0 |
|  | Oral candidiasis | 0 | 0.0 | 1 | 8.3 | 0 | 0.0 | 0 | 0.0 | 1 | 4.0 |
|  | Paronychia | 0 | 0.0 | 0 | 0.0 | 1 | 16.7 | 0 | 0.0 | 1 | 4.0 |
|  | Pharyngitis | 0 | 0.0 | 1 | 8.3 | 0 | 0.0 | 0 | 0.0 | 1 | 4.0 |
|  | Pneumonia fungal | 0 | 0.0 | 1 | 8.3 | 0 | 0.0 | 0 | 0.0 | 1 | 4.0 |
|  | Puncture site infection | 1 | 33.3 | 0 | 0.0 | 0 | 0.0 | 0 | 0.0 | 1 | 4.0 |
|  | Urinary tract infection | 0 | 0.0 | 1 | 8.3 | 0 | 0.0 | 0 | 0.0 | 1 | 4.0 |
| Investigations |  | 1 | 33.3 | 6 | 50.0 | 2 | 33.3 | 1 | 25.0 | 10 | 40.0 |
|  | C-reactive protein increased | 1 | 33.3 | 2 | 16.7 | 1 | 16.7 | 0 | 0.0 | 4 | 16.0 |
|  | Blood creatinine increased | 0 | 0.0 | 1 | 8.3 | 1 | 16.7 | 0 | 0.0 | 2 | 8.0 |
|  | Blood bilirubin increased | 0 | 0.0 | 1 | 8.3 | 0 | 0.0 | 0 | 0.0 | 1 | 4.0 |
|  | Gamma-glutamyltransferase increased | 0 | 0.0 | 1 | 8.3 | 0 | 0.0 | 0 | 0.0 | 1 | 4.0 |
|  | Nutritional condition abnormal | 0 | 0.0 | 1 | 8.3 | 0 | 0.0 | 0 | 0.0 | 1 | 4.0 |
|  | Platelet count decreased | 0 | 0.0 | 0 | 0.0 | 0 | 0.0 | 1 | 25.0 | 1 | 4.0 |
|  | Procalcitonin increased | 0 | 0.0 | 0 | 0.0 | 1 | 16.7 | 0 | 0.0 | 1 | 4.0 |
|  | Weight decreased | 0 | 0.0 | 1 | 8.3 | 0 | 0.0 | 0 | 0.0 | 1 | 4.0 |
| Nervous system disorders |  | 2 | 66.7 | 5 | 41.7 | 1 | 16.7 | 2 | 50.0 | 10 | 40.0 |
|  | Dizziness | 0 | 0.0 | 4 | 33.3 | 0 | 0.0 | 1 | 25.0 | 5 | 20.0 |
|  | Headache | 1 | 33.3 | 0 | 0.0 | 1 | 16.7 | 0 | 0.0 | 2 | 8.0 |
|  | Syncope | 1 | 33.3 | 0 | 0.0 | 0 | 0.0 | 1 | 25.0 | 2 | 8.0 |
|  | Cognitive disorder | 0 | 0.0 | 1 | 8.3 | 0 | 0.0 | 0 | 0.0 | 1 | 4.0 |
|  | Intracranial haematoma | 0 | 0.0 | 1 | 8.3 | 0 | 0.0 | 0 | 0.0 | 1 | 4.0 |
|  | Seizure | 1 | 33.3 | 0 | 0.0 | 0 | 0.0 | 0 | 0.0 | 1 | 4.0 |
| Respiratory, thoracic and mediastinal disorders |  | 1 | 33.3 | 6 | 50.0 | 0 | 0.0 | 2 | 50.0 | 9 | 36.0 |
|  | Epistaxis | 0 | 0.0 | 6 | 50.0 | 0 | 0.0 | 0 | 0.0 | 6 | 24.0 |
|  | Dyspnoea | 1 | 33.3 | 2 | 16.7 | 0 | 0.0 | 1 | 25.0 | 4 | 16.0 |
|  | Cough | 0 | 0.0 | 2 | 16.7 | 0 | 0.0 | 0 | 0.0 | 2 | 8.0 |
|  | Hypoxia | 0 | 0.0 | 1 | 8.3 | 0 | 0.0 | 1 | 25.0 | 2 | 8.0 |
|  | Pleural effusion | 0 | 0.0 | 0 | 0.0 | 0 | 0.0 | 1 | 25.0 | 1 | 4.0 |
|  | Productive cough | 0 | 0.0 | 1 | 8.3 | 0 | 0.0 | 0 | 0.0 | 1 | 4.0 |
| Injury, poisoning and procedural complications |  | 1 | 33.3 | 3 | 25.0 | 1 | 16.7 | 2 | 50.0 | 7 | 28.0 |
|  | Fall | 1 | 33.3 | 0 | 0.0 | 0 | 0.0 | 2 | 50.0 | 3 | 12.0 |
|  | Skin laceration | 0 | 0.0 | 2 | 16.7 | 1 | 16.7 | 0 | 0.0 | 3 | 12.0 |
|  | Facial bones fracture | 0 | 0.0 | 1 | 8.3 | 0 | 0.0 | 0 | 0.0 | 1 | 4.0 |
|  | Post transfusion purpura | 0 | 0.0 | 1 | 8.3 | 0 | 0.0 | 0 | 0.0 | 1 | 4.0 |
|  | Subdural haematoma | 0 | 0.0 | 1 | 8.3 | 0 | 0.0 | 0 | 0.0 | 1 | 4.0 |
|  | Tooth injury | 0 | 0.0 | 1 | 8.3 | 0 | 0.0 | 0 | 0.0 | 1 | 4.0 |
| Psychiatric disorders |  | 0 | 0.0 | 2 | 16.7 | 3 | 50.0 | 1 | 25.0 | 6 | 24.0 |
|  | Confusional state | 0 | 0.0 | 0 | 0.0 | 2 | 33.3 | 1 | 25.0 | 3 | 12.0 |
|  | Delirium | 0 | 0.0 | 0 | 0.0 | 1 | 16.7 | 0 | 0.0 | 1 | 4.0 |
|  | Disorientation | 0 | 0.0 | 1 | 8.3 | 0 | 0.0 | 0 | 0.0 | 1 | 4.0 |
|  | Insomnia | 0 | 0.0 | 1 | 8.3 | 0 | 0.0 | 0 | 0.0 | 1 | 4.0 |
| Skin and subcutaneous tissue disorders |  | 1 | 33.3 | 3 | 25.0 | 1 | 16.7 | 1 | 25.0 | 6 | 24.0 |
|  | Pruritus | 0 | 0.0 | 1 | 8.3 | 0 | 0.0 | 1 | 25.0 | 2 | 8.0 |
|  | Decubitus ulcer | 0 | 0.0 | 1 | 8.3 | 0 | 0.0 | 0 | 0.0 | 1 | 4.0 |
|  | Erythema | 0 | 0.0 | 0 | 0.0 | 1 | 16.7 | 0 | 0.0 | 1 | 4.0 |
|  | Petechiae | 0 | 0.0 | 1 | 8.3 | 0 | 0.0 | 0 | 0.0 | 1 | 4.0 |
|  | Rash | 0 | 0.0 | 0 | 0.0 | 1 | 16.7 | 0 | 0.0 | 1 | 4.0 |
|  | Skin exfoliation | 0 | 0.0 | 1 | 8.3 | 0 | 0.0 | 0 | 0.0 | 1 | 4.0 |
|  | Skin fissures | 1 | 33.3 | 0 | 0.0 | 0 | 0.0 | 0 | 0.0 | 1 | 4.0 |
| Vascular disorders |  | 1 | 33.3 | 3 | 25.0 | 1 | 16.7 | 1 | 25.0 | 6 | 24.0 |
|  | Hypotension | 1 | 33.3 | 2 | 16.7 | 1 | 16.7 | 0 | 0.0 | 4 | 16.0 |
|  | Haematoma | 0 | 0.0 | 2 | 16.7 | 0 | 0.0 | 0 | 0.0 | 2 | 8.0 |
|  | Thrombophlebitis | 0 | 0.0 | 0 | 0.0 | 0 | 0.0 | 1 | 25.0 | 1 | 4.0 |
| Musculoskeletal and connective tissue disorders |  | 1 | 33.3 | 4 | 33.3 | 0 | 0.0 | 0 | 0.0 | 5 | 20.0 |
|  | Arthralgia | 1 | 33.3 | 2 | 16.7 | 0 | 0.0 | 0 | 0.0 | 3 | 12.0 |
|  | Bone pain | 0 | 0.0 | 1 | 8.3 | 0 | 0.0 | 0 | 0.0 | 1 | 4.0 |
|  | Flank pain | 0 | 0.0 | 1 | 8.3 | 0 | 0.0 | 0 | 0.0 | 1 | 4.0 |
|  | Joint effusion | 0 | 0.0 | 1 | 8.3 | 0 | 0.0 | 0 | 0.0 | 1 | 4.0 |
|  | Muscular weakness | 0 | 0.0 | 1 | 8.3 | 0 | 0.0 | 0 | 0.0 | 1 | 4.0 |
| Metabolism and nutrition disorders |  | 0 | 0.0 | 3 | 25.0 | 1 | 16.7 | 0 | 0.0 | 4 | 16.0 |
|  | Decreased appetite | 0 | 0.0 | 1 | 8.3 | 1 | 16.7 | 0 | 0.0 | 2 | 8.0 |
|  | Hyperkalaemia | 0 | 0.0 | 1 | 8.3 | 0 | 0.0 | 0 | 0.0 | 1 | 4.0 |
|  | Hypokalaemia | 0 | 0.0 | 1 | 8.3 | 0 | 0.0 | 0 | 0.0 | 1 | 4.0 |
|  | Hyponatraemia | 0 | 0.0 | 1 | 8.3 | 0 | 0.0 | 0 | 0.0 | 1 | 4.0 |
| Renal and urinary disorders |  | 0 | 0.0 | 2 | 16.7 | 1 | 16.7 | 0 | 0.0 | 3 | 12.0 |
|  | Acute kidney injury | 0 | 0.0 | 0 | 0.0 | 1 | 16.7 | 0 | 0.0 | 1 | 4.0 |
|  | Haematinuria | 0 | 0.0 | 1 | 8.3 | 0 | 0.0 | 0 | 0.0 | 1 | 4.0 |
|  | Renal failure | 0 | 0.0 | 1 | 8.3 | 0 | 0.0 | 0 | 0.0 | 1 | 4.0 |
| Cardiac disorders |  | 0 | 0.0 | 1 | 8.3 | 0 | 0.0 | 1 | 25.0 | 2 | 8.0 |
|  | Bradycardia | 0 | 0.0 | 0 | 0.0 | 0 | 0.0 | 1 | 25.0 | 1 | 4.0 |
|  | Pericardial effusion | 0 | 0.0 | 1 | 8.3 | 0 | 0.0 | 0 | 0.0 | 1 | 4.0 |
| Ear and labyrinth disorders |  | 0 | 0.0 | 0 | 0.0 | 0 | 0.0 | 1 | 25.0 | 1 | 4.0 |
|  | Vertigo | 0 | 0.0 | 0 | 0.0 | 0 | 0.0 | 1 | 25.0 | 1 | 4.0 |
| Immune system disorders |  | 0 | 0.0 | 1 | 8.3 | 0 | 0.0 | 0 | 0.0 | 1 | 4.0 |
|  | Alloimmunisation | 0 | 0.0 | 1 | 8.3 | 0 | 0.0 | 0 | 0.0 | 1 | 4.0 |
| Reproductive system and breast disorders |  | 0 | 0.0 | 1 | 8.3 | 0 | 0.0 | 0 | 0.0 | 1 | 4.0 |
|  | Vaginal haemorrhage | 0 | 0.0 | 1 | 8.3 | 0 | 0.0 | 0 | 0.0 | 1 | 4.0 |

TCP dose level: Level 1 = 20 mg, Level 2 = 40 mg, Level 3 = 60mg, Level 4 = 80 mg

| **Supplementary Table 2:** Incidence of AEs by System Organ Class (SOC), and Preferred Term (PT): All serious adverse events |
| --- |

| *Supplementary Table 2: Incidence of AEs by System Organ Class (SOC), and Preferred Term (PT): All serious adverse events* | | | | | | | | | | | |
| --- | --- | --- | --- | --- | --- | --- | --- | --- | --- | --- | --- |
|  | | *Level achieved* | | | | | | | |  | |
|  | | *Level 1* | | *Level 2* | | *Level 3* | | *Level 4* | | *Total* | |
| *System organ class* | *Preferred term* | *No.* | *Total %* | *No.* | *Total %* | *No.* | *Total %* | *No.* | *Total %* | *No.* | *Total %* |
| Total number of patients |  | 3 | 100.0 | 12 | 100.0 | 6 | 100.0 | 4 | 100.0 | 25 | 100.0 |
| Number of patients with at least one SAE |  | 2 | 66.7 | 9 | 75.0 | 4 | 66.7 | 4 | 100.0 | 19 | 76.0 |
| Infections and infestations |  | 2 | 66.7 | 7 | 58.3 | 1 | 16.7 | 2 | 50.0 | 12 | 48.0 |
|  | Pneumonia | 0 | 0.0 | 3 | 25.0 | 0 | 0.0 | 1 | 25.0 | 4 | 16.0 |
|  | Sepsis | 1 | 33.3 | 1 | 8.3 | 0 | 0.0 | 0 | 0.0 | 2 | 8.0 |
|  | Bacterial infection | 0 | 0.0 | 0 | 0.0 | 1 | 16.7 | 0 | 0.0 | 1 | 4.0 |
|  | Device related infection | 1 | 33.3 | 0 | 0.0 | 0 | 0.0 | 0 | 0.0 | 1 | 4.0 |
|  | Escherichia infection | 0 | 0.0 | 1 | 8.3 | 0 | 0.0 | 0 | 0.0 | 1 | 4.0 |
|  | Escherichia sepsis | 0 | 0.0 | 1 | 8.3 | 0 | 0.0 | 0 | 0.0 | 1 | 4.0 |
|  | Haematoma infection | 0 | 0.0 | 1 | 8.3 | 0 | 0.0 | 0 | 0.0 | 1 | 4.0 |
|  | Neutropenic infection | 0 | 0.0 | 0 | 0.0 | 0 | 0.0 | 1 | 25.0 | 1 | 4.0 |
|  | Neutropenic sepsis | 0 | 0.0 | 1 | 8.3 | 0 | 0.0 | 0 | 0.0 | 1 | 4.0 |
|  | Pneumonia fungal | 0 | 0.0 | 1 | 8.3 | 0 | 0.0 | 0 | 0.0 | 1 | 4.0 |
|  | Puncture site infection | 1 | 33.3 | 0 | 0.0 | 0 | 0.0 | 0 | 0.0 | 1 | 4.0 |
|  | Urinary tract infection | 0 | 0.0 | 1 | 8.3 | 0 | 0.0 | 0 | 0.0 | 1 | 4.0 |
| Blood and lymphatic system disorders |  | 0 | 0.0 | 1 | 8.3 | 1 | 16.7 | 2 | 50.0 | 4 | 16.0 |
|  | Febrile neutropenia | 0 | 0.0 | 1 | 8.3 | 0 | 0.0 | 2 | 50.0 | 3 | 12.0 |
|  | Pancytopenia | 0 | 0.0 | 0 | 0.0 | 1 | 16.7 | 0 | 0.0 | 1 | 4.0 |
| Gastrointestinal disorders |  | 0 | 0.0 | 0 | 0.0 | 2 | 33.3 | 1 | 25.0 | 3 | 12.0 |
|  | Gastrointestinal haemorrhage | 0 | 0.0 | 0 | 0.0 | 0 | 0.0 | 1 | 25.0 | 1 | 4.0 |
|  | Lower gastrointestinal haemorrhage | 0 | 0.0 | 0 | 0.0 | 1 | 16.7 | 0 | 0.0 | 1 | 4.0 |
|  | Vomiting | 0 | 0.0 | 0 | 0.0 | 1 | 16.7 | 0 | 0.0 | 1 | 4.0 |
| Injury, poisoning and procedural complications |  | 0 | 0.0 | 2 | 16.7 | 1 | 16.7 | 0 | 0.0 | 3 | 12.0 |
|  | Post transfusion purpura | 0 | 0.0 | 1 | 8.3 | 0 | 0.0 | 0 | 0.0 | 1 | 4.0 |
|  | Skin laceration | 0 | 0.0 | 0 | 0.0 | 1 | 16.7 | 0 | 0.0 | 1 | 4.0 |
|  | Subdural haematoma | 0 | 0.0 | 1 | 8.3 | 0 | 0.0 | 0 | 0.0 | 1 | 4.0 |
| Nervous system disorders |  | 1 | 33.3 | 1 | 8.3 | 0 | 0.0 | 0 | 0.0 | 2 | 8.0 |
|  | Intracranial haematoma | 0 | 0.0 | 1 | 8.3 | 0 | 0.0 | 0 | 0.0 | 1 | 4.0 |
|  | Syncope | 1 | 33.3 | 0 | 0.0 | 0 | 0.0 | 0 | 0.0 | 1 | 4.0 |
| General disorders and administration site conditions |  | 0 | 0.0 | 0 | 0.0 | 1 | 16.7 | 0 | 0.0 | 1 | 4.0 |
|  | General physical health deterioration | 0 | 0.0 | 0 | 0.0 | 1 | 16.7 | 0 | 0.0 | 1 | 4.0 |
| Investigations |  | 0 | 0.0 | 1 | 8.3 | 0 | 0.0 | 0 | 0.0 | 1 | 4.0 |
|  | Gamma-glutamyltransferase increased | 0 | 0.0 | 1 | 8.3 | 0 | 0.0 | 0 | 0.0 | 1 | 4.0 |
| Metabolism and nutrition disorders |  | 0 | 0.0 | 1 | 8.3 | 0 | 0.0 | 0 | 0.0 | 1 | 4.0 |
|  | Hyponatraemia | 0 | 0.0 | 1 | 8.3 | 0 | 0.0 | 0 | 0.0 | 1 | 4.0 |
| Psychiatric disorders |  | 0 | 0.0 | 0 | 0.0 | 1 | 16.7 | 0 | 0.0 | 1 | 4.0 |
|  | Confusional state | 0 | 0.0 | 0 | 0.0 | 1 | 16.7 | 0 | 0.0 | 1 | 4.0 |

TCP dose level: Level 1 = 20 mg, Level 2 = 40 mg, Level 3 = 60mg, Level 4 = 80 mg
